# Anti-EGFR Antibody–Drug Conjugate Carrying an Inhibitor Targeting CDK Restricts Triple-Negative Breast Cancer Growth

**DOI:** 10.1158/1078-0432.CCR-23-3110

**Published:** 2024-05-21

**Authors:** Anthony Cheung, Alicia M. Chenoweth, Annelie Johansson, Roman Laddach, Naomi Guppy, Jennifer Trendell, Benjamina Esapa, Antranik Mavousian, Blanca Navarro-Llinas, Syed Haider, Pablo Romero-Clavijo, Ricarda M. Hoffmann, Paolo Andriollo, Khondaker M. Rahman, Paul Jackson, Sophia Tsoka, Sheeba Irshad, Ioannis Roxanis, Anita Grigoriadis, David E. Thurston, Christopher J. Lord, Andrew N. J. Tutt, Sophia N. Karagiannis

**Affiliations:** 1 Breast Cancer Now Research Unit, School of Cancer and Pharmaceutical Sciences, King’s College London, Guy’s Cancer Centre, London, United Kingdom.; 2 St. John’s Institute of Dermatology, School of Basic and Medical Biosciences & KHP Centre for Translational Medicine, King’s College London, Guy’s Hospital, London, United Kingdom.; 3 Cancer Bioinformatics, School of Cancer and Pharmaceutical Sciences, King’s College London, Guy’s Cancer Centre, London, United Kingdom.; 4 Department of Informatics, Faculty of Natural, Mathematical and Engineering Sciences, King’s College London, London, United Kingdom.; 5 The Breast Cancer Now Toby Robins Research Centre, The Institute of Cancer Research, London, United Kingdom.; 6 Institute of Pharmaceutical Science, School of Cancer and Pharmaceutical Sciences, King’s College London, London, United Kingdom.

## Abstract

**Purpose:**

Anti-EGFR antibodies show limited response in breast cancer, partly due to activation of compensatory pathways. Furthermore, despite the clinical success of cyclin-dependent kinase (CDK) 4/6 inhibitors in hormone receptor–positive tumors, aggressive triple-negative breast cancers (TNBC) are largely resistant due to CDK2/cyclin E expression, whereas free CDK2 inhibitors display normal tissue toxicity, limiting their therapeutic application. A cetuximab-based antibody drug conjugate (ADC) carrying a CDK inhibitor selected based on oncogene dysregulation, alongside patient subgroup stratification, may provide EGFR-targeted delivery.

**Experimental Design:**

Expressions of G1/S-phase cell cycle regulators were evaluated alongside EGFR in breast cancer. We conjugated cetuximab with CDK inhibitor SNS-032, for specific delivery to EGFR-expressing cells. We assessed ADC internalization and its antitumor functions *in vitro* and in orthotopically grown basal-like/TNBC xenografts.

**Results:**

Transcriptomic (6,173 primary, 27 baseline, and matched post-chemotherapy residual tumors), single-cell RNA sequencing (150,290 cells, 27 treatment-naïve tumors), and spatial transcriptomic (43 tumor sections, 22 TNBCs) analyses confirmed expression of CDK2 and its cyclin partners in basal-like/TNBCs, associated with EGFR. Spatiotemporal live-cell imaging and super-resolution confocal microscopy demonstrated ADC colocalization with late lysosomal clusters. The ADC inhibited cell cycle progression, induced cytotoxicity against high EGFR-expressing tumor cells, and bystander killing of neighboring EGFR-low tumor cells, but minimal effects on immune cells. Despite carrying a small molar fraction (1.65%) of the SNS-032 inhibitor, the ADC restricted EGFR-expressing spheroid and cell line/patient-derived xenograft tumor growth.

**Conclusions:**

Exploiting EGFR overexpression, and dysregulated cell cycle in aggressive and treatment-refractory tumors, a cetuximab–CDK inhibitor ADC may provide selective and efficacious delivery of cell cycle–targeted agents to basal-like/TNBCs, including chemotherapy-resistant residual disease.

**Translational Relevance**

Approved antibody drug conjugates (ADC) carry classical broadly toxic payloads targeting DNA or microtubules. Investigating intracellular pathways, which aggressive and drug-resistant cancers depend on, may offer therapeutic approaches that combine a tumor-selective antibody and a payload targeting dysregulated cancer mechanism. Triple-negative breast cancers (TNBC) are largely resistant to cell cycle inhibitors against cyclin-dependent kinase (CDK) 4/6 because of CDK2/cyclin E expression; however, free CDK2 inhibitors display toxicity to normal tissues. We provide evidence of the association between EGFR and the G1/S-phase cell cycle regulators CDK2/cyclin E in basal-like/TNBCs, including chemotherapy-resistant disease, and develop a cetuximab-based ADC, conjugated with a CDK inhibitor, for drug delivery to EGFR-expressing cancer. Despite carrying a small fraction of the inhibitor dose needed to exert antitumor effects, the ADC restricted TNBC growth, including in a patient-derived xenograft model. We introduce a next-generation ADC strategy targeting surface antigen and druggable oncogenic signaling activity in selected patient cohorts with limited treatment options.

## Introduction

Triple-negative breast cancer (TNBC) comprises a heterogeneous disease group defined by lack of estrogen receptor (ER), progesterone receptor (PR), and HER2 expression and is often associated with increased genomic instability, high mitotic rates and poor prognosis (1, 2). Historically, treatment options were limited to surgery, adjuvant chemotherapy, and radiotherapy; however, recent advancements have led to the approval of several targeted therapies. These include the antiprogrammed death-ligand 1 (PD-L1) antibody atezolizumab as immunotherapy in combination with nab-paclitaxel chemotherapy for advanced-stage TNBC (3) and olaparib for adjuvant treatment of patients with deleterious or suspected deleterious germline BRCA variants (4). The anti-TROP2 antibody drug conjugate (ADC) sacituzumab govitecan, with sacituzumab coupled to topoisomerase I inhibitor SN38, was recently approved for the treatment of relapsed or metastatic TNBCs, highlighting the promise of ADC therapies (5). However, challenges remain for patients with intrinsic or acquired resistance-driving mechanisms (6–8). Determining the diverse molecular characteristics of TNBCs to select targeted therapies may help identify patient groups most likely to derive benefit from new treatment approaches or combinations.

Based on gene expression (9), EGFR has been investigated to be a targetable cancer-associated marker that may serve to define patient subgroups potentially suitable for EGFR-directed therapy approaches. EGFR enhances cancer cell proliferation and survival, and its overexpression is common in TNBCs, ranging from 36% to 89% of cases (10, 11). Potential EGFR-targeting therapies, such as monoclonal antibodies and tyrosine kinase inhibitors, directed to its complex signaling network have been explored in clinical trials (12). Despite clinical success in colorectal cancer and head and neck cancer (13, 14), the anti-EGFR antibodies cetuximab and panitumumab have shown limited response rates in TNBCs and unselected patient populations (15, 16), likely due to activation of alternative compensatory pathways and inter-/intra-tumoral heterogeneity in EGFR expression (17).

Recent advancements in the development and approval of ADCs have led to renewed interest in EGFR (18, 19). ADCs combine the specificity of an antibody with a potent cytotoxic warhead. These properties allow selective recognition and killing of malignant cells, whereas, in principle, sparing healthy cells, depending on the normal tissue distribution of the target, and avoiding systemic exposure to payloads (20). Conjugation of an inhibitor to cetuximab could potentially improve its therapeutic index, since the antitumor effect is not solely dependent on the inhibition of downstream EGFR signaling pathways for which intrinsic or acquired drug resistance is reported (17, 21).

Preclinical antitumor activities of EGFR-targeted ADCs bearing auristatin payloads have been evaluated in solid tumors (18, 19) and have thus far shown manageable safety profiles in two clinical trials (22, 23). These ADCs carry classical payloads targeting DNA or microtubules, which in principle could impact any proliferating cell and therefore normal organ function, limiting selective antitumor effects and therapeutic window. However, novel combinations of anti-EGFR antibodies linked to inhibitors targeting dysregulated cancer cell–associated pathways might offer advantages with regard to (i) specific delivery of the inhibitor to tumor cells with a likely perturbed cell cycle transition axis and (ii) systemic cytotoxicity reduction.

Despite the clinical application of selective cyclin-dependent kinase (CDK) 4/6 inhibitors (palbociclib, ribociclib, and abemaciclib) in hormone receptor–positive breast cancer, the CDK2/cyclin E complex that drives G1/S-phase transition is dysregulated in TNBCs and contributes to cell cycle–specific drug resistance (24). This evasion mechanism may present a potentially druggable pathway. Encouraging results have been reported for inhibitors targeting cell cycle alteration mechanisms in breast cancers (24, 25). However, broadly active CDK inhibitors administered alone have displayed normal tissue toxicity in clinical testing, limiting their therapeutic potential. Therefore, a tumor cell–targeting (i.e., ADC) approach for these inhibitors may enhance their therapeutic window, offering an opportunity to direct these more specifically to cancer cells.

In this study, we examined a combined targeting approach against EGFR and G1/S-phase cell cycle molecules by generating an anti-EGFR ADC conjugated with a CDK inhibitor to directly attack EGFR-expressing cells and their microenvironment. We ascertained EGFR expression and cyclin A, cyclin E, and CDK2 levels in breast cancers by bulk, single-cell, and spatial transcriptomic analyses. We assessed the potential antitumor functions of an anti-EGFR ADC, with cetuximab stochastically conjugated to a CDK inhibitor SNS-032, known to have selective inhibition of CDK2/7/9 over CDK4/6 (25). We conducted a spatiotemporal analysis of ADC internalization by live-cell imaging microscopy. In cell-based assays, we interrogated the effect of this ADC on cell cycle and cellular viability *in vitro* and on orthotopically grown human TNBC xenografts in the mammary fat pads of immunodeficient mice.

## Materials and Methods

### Ethics

Human samples were collected with informed written consent, in accordance with the Helsinki Declaration, and the study design was approved by London-Chelsea Research Ethics (REC number: 13/LO/1248, IRAS ID 131133). Patients were staged and classified according to the TNM Classification of Malignant Tumors accepted by the Union for International Cancer Control.

### Gene expression data of human breast cancers

Detailed descriptions of the human female cancer cohorts, including TNBC-enriched King’s College London (KCL) Guy’s Hospital (Guy’s) cohort (*n* = 177), Sweden Cancerome Analysis Network-Breast cohort (*n* = 3,273), METABRIC cohort (*n* = 1,380), The Cancer Genome Atlas (TCGA) breast cancer cohort (*n* = 1,084), International Cancer Genome Consortium (ICGC) cohort (*n* = 259; refs. 26–30), matched baseline–residual TNBC cohort (*n* = 27; ref. 31), single-cell RNA sequencing (scRNA-seq) cohort (*n* = 27; ref. 32), and spatial transcriptomic cohort (*n* = 16; ref. 33) have been published previously. Details of the cohort samples are summarized in Supplementary Table S1.

**Figure 1.**
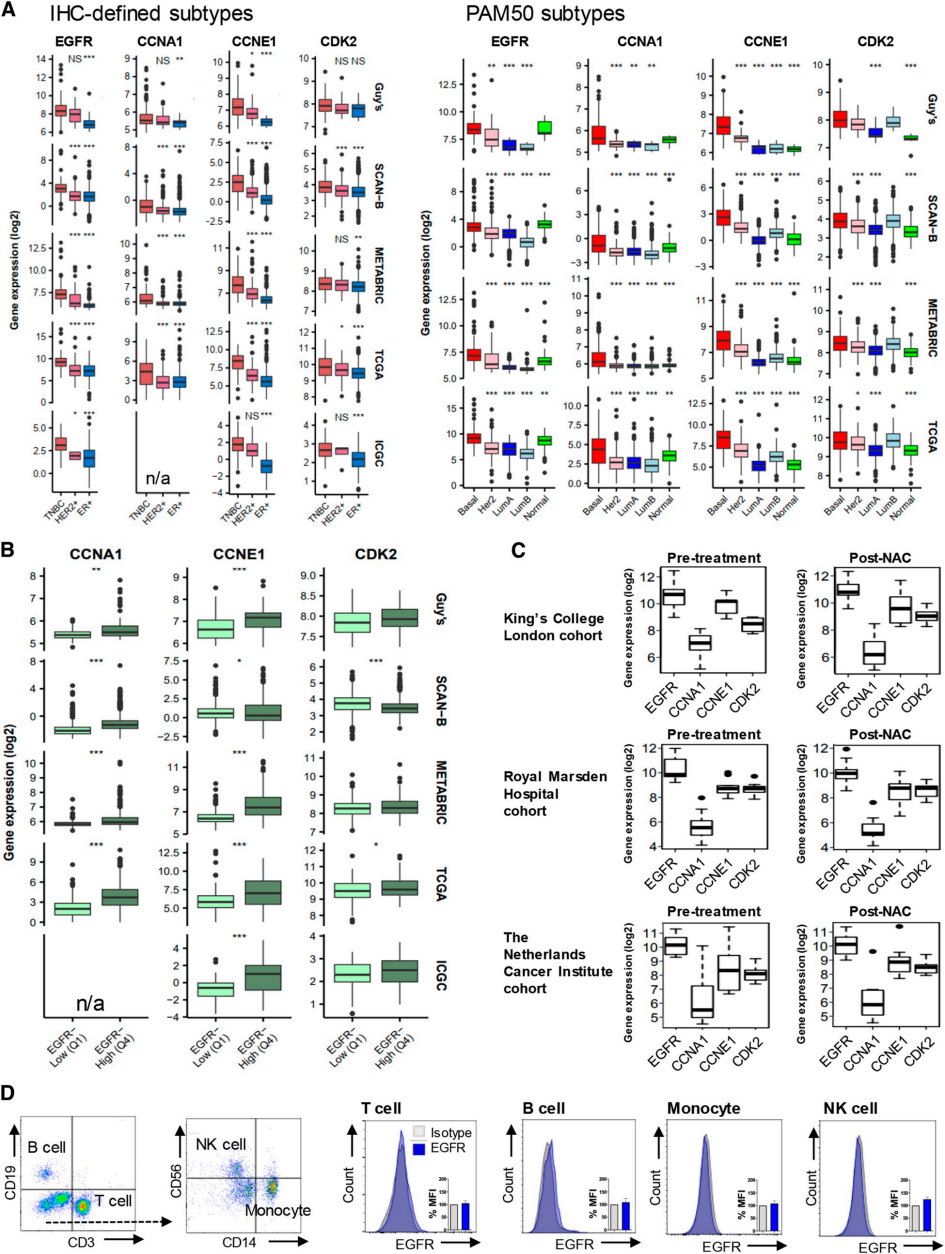
Basal-like/TNBC is associated with upregulated EGFR and G1/S-phase cell cycle genes. **A,** Gene expression analysis of *EGFR*, *CCNA1*, *CCNE1*, and *CDK2* was stratified according to IHC-defined receptor status from five published databases, *n* = 6,173 primary tumors: Guy’s (TNBC vs. HER2+ vs. ER+, *n* = 131 vs. 32 vs. 14), Sweden Cancerome Analysis Network—Breast (SCAN-B) (*n* = 165 vs. 420 vs. 2,425), METABRIC (*n* = 101 vs. 117 vs. 347), The Cancer Genome Atlas (TCGA) (*n* = 112 vs. 158 vs. 426), International Cancer Genome Consortium (ICGC) (*n* = 73 vs. 4 vs. 182; refs. 26–30).* CCNA1* was not included in the ICGC cohort, therefore marked as unavailable (n/a). The cohorts were analyzed by PAM50 classification [Basal-like (Basal), HER2, luminal A (LumA), luminal B (LumB), normal-like (Normal): Guy’s (*n* = 95, 28, 11, 10, 7), SCAN-B (*n* = 339, 327, 1657, 729, 221), METABRIC (*n* = 237, 181, 483, 383, 93), and TCGA (*n* = 232, 153, 345, 263, 91)]. All *P* values are compared against TNBC or Basal-like subtype. **B,** Expression of G1/S-phase genes were compared between low and high EGFR-expressing samples based on quartile ranges of gene expression values (EGFR-low, first quartile Q1; EGFR-high, fourth quartile Q4). Median-centered gene expression log_2_ values are shown. *P* values determined using Mann–Whitney U test. **C,** Gene expression was compared between matched pre-treatment and residual TNBC samples [KCL cohort: (*n* = 8); Royal Marsden Hospital cohort (*n* = 9); The Netherlands Cancer Institute cohort (*n* = 10)]. Cohort details: Supplementary Table S1. **D,** EGFR expression measured by flow cytometry in peripheral blood mononuclear cells (PBMC; *n* = 3) following Fc-receptor blocking solution.

Clinicopathological and gene expression data were extracted from the publications and compared between IHC-defined subtypes based on breast pathology evaluation of ER, PR, and HER2 receptors or compared between PAM50 subtypes based on the expression of 50 genes to subclassify breast cancers into five distinct subtypes, i.e., Basal-like, HER2-enriched, Luminal A, Luminal B, and Normal-like breast cancer (25). PAM50 information was not included in the ICGC cohort. Data from the bulk transcriptomic cohorts were not merged and analyzed separately. Classification of EFGR-high or EGFR-low was divided into quartiles for all patient cohorts, comparing the highest quartile (Q4) against the lowest quartile (Q1). All statistical analysis and respective data plots were generated in R version 4.2.2 using several CRAN packages (http://cran.rproject.org/). Codes for Fig. 1A and B are available at Github: https://github.com/annelieewa/anti-EGFR-ADC/. For the matched baseline–residual TNBC cohort, gene expression was compared between pre-treatment and post-neoadjuvant chemotherapy–resistant (post-NAC-resistant) TNBC samples previously treated with sequential anthracyclines, taxane or platinum chemotherapy [KCL: (*n* = 8); Royal Marsden Hospital (*n* = 9); The Netherlands Cancer Institute (*n* = 10; ref. 31)]. The gene expression of EGFR in cell line models was analyzed using the online database Cancer Cell Line Encyclopedia (https://portals.broadinstitute.org/ccle).

scRNA-seq data were obtained from the publicly available dataset GSE161529 (32). Pre-processed, batch-corrected datasets in the format of Seurat objects (34) created by the original authors were used to visualize gene expression levels of *EGFR*, *CCNE1*, and *CDK2* using the same R package. Combined scRNA-seq transcriptomes of total 150,290 cells from 27 untreated primary tumors (TNBC, *n* = 8 samples, 54,819 cells; HER2+, *n* = 6 samples, 31,917 cells; ER+, *n* = 13 samples, 63,554 cells) were analyzed by dimensionality reduction of the *t*-distributed stochastic neighbor embedding (t-SNE) algorithm to project all cell events from each set of samples into two-dimensional maps. Cell population identification was conducted with hierarchical clustering. The raw expression data from each Seurat object were binarized and cells were further filtered to include the epithelial cell adhesion molecule (*EpCAM*)+ subsets (tumor cell populations) for downstream analysis, and marker gene expression levels were colored by intensity.

For spatial transcriptomic analysis, the publicly available Visium dataset GSE210616 (33) was used to investigate levels and co-occurrence of *EpCAM*, *EGFR*, *CCNE1*, and *CDK2* in tissue spots. The raw output from SpaceRanger, available on Gene Expression Omnibus (GEO; RRID:SCR_005012), was used to generate Seurat objects. Data were analyzed using the Seurat packages and normalized using the SCTransform function to visualize gene levels across spots. Spatial mapping of gene expression was performed in *EpCAM*+ cell clusters of 43 tumor sections from 22 patients with TNBC, where 26 sections were from 13 treatment-naïve patients and 17 sections were from nine post-NAC residual disease patients. Raw counts from all available tissue slices were binarized into zero and nonzero counts to quantify the spatial co-occurrence of genes of interest, after dividing the dataset into patients before and after treatment per original demographics.

**Figure 2.**
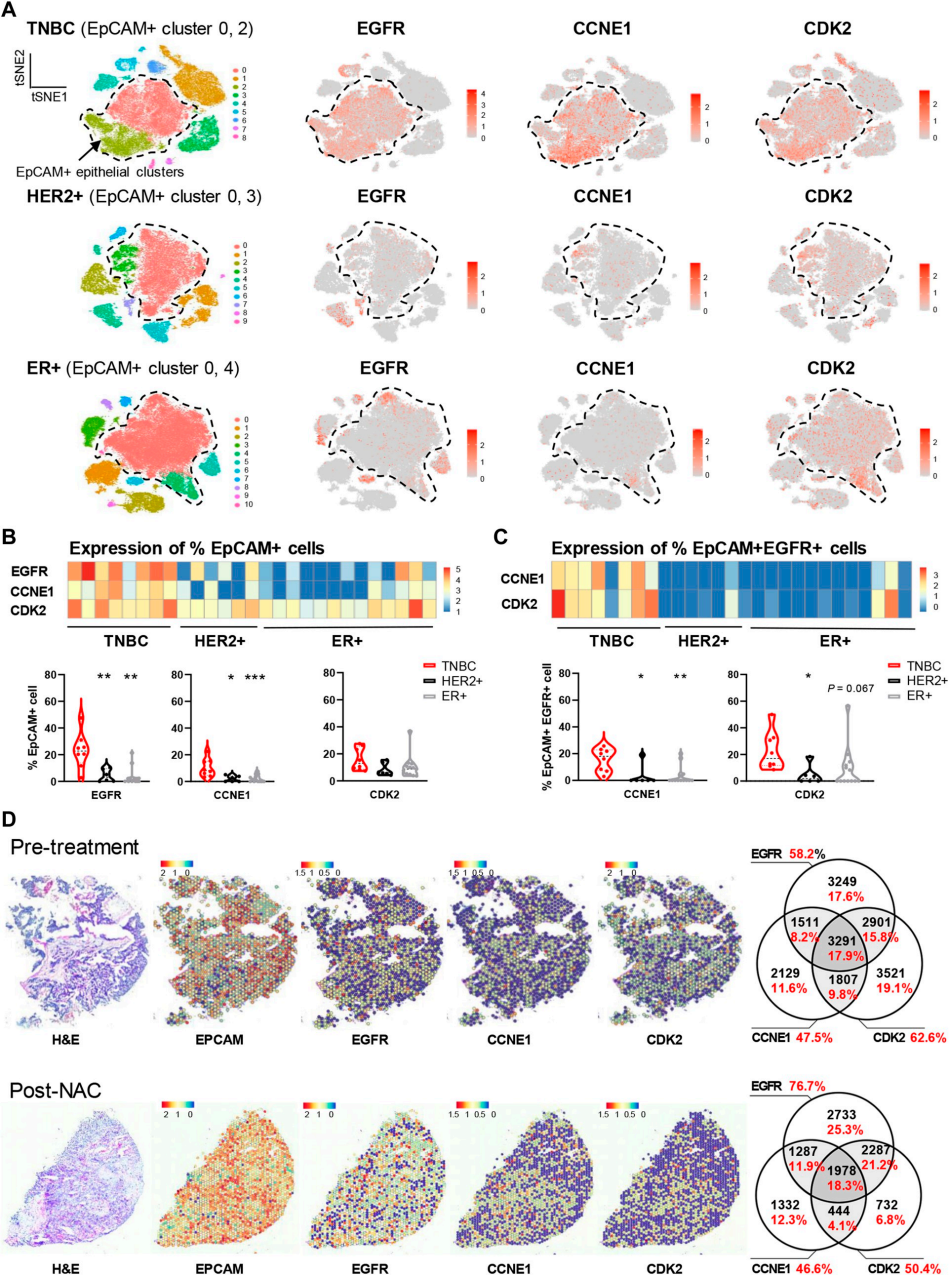
Single-cell RNA sequencing and spatial transcriptomic analyses reveal cellular and spatial co-expression of EGFR with CDK2/cyclin E in TNBCs. **A,** Dimensionality reduction *t*-distributed stochastic neighbor embedding (*t*-SNE) map of combined scRNA-seq transcriptomes of 150,290 cells from 27 untreated primary tumors (TNBC, *n* = 8 samples, 54,819 cells; HER2+, *n* = 6 samples, 31,917 cells; ER+, *n* = 13 samples, 63,554 cells) colored by cell cluster (32). *EpCAM* expression revealed two prominent *EpCAM*+ tumor epithelial clusters in each cancer subtype (dotted lines). *t*-SNE plots showing expression levels of *EGFR*, *CCNE1*, and *CDK2* genes for the same clusters. Red: high expression, gray: not detected. Overall expression of *CCNA1* was too low and was excluded from the analysis. **B,** Analyses of the transcriptomic data were conducted to evaluate expression of *EGFR*, *CCNE1*, and *CDK2* genes per patient and for each patient cohort (TNBC, HER2+, and ER+). Heatmap (top) with color boxes indicating normalized expression level; each column represents a patient tumor. Quantitative analysis (bottom) was calculated by the number of cells expressing *EGFR*, *CCNE1*, or *CDK2*, in proportion to *EpCAM*+ cells. **C,** Same analysis was performed on *EpCAM*+ *EGFR*+ cells for the expression of *CCNE1* and *CDK2*. *P* values determined by two-tailed unpaired *t* test against TNBC subtype. **D,** Spatial transcriptomic analysis of *EGFR*,* CCNE1*, and* CDK2 *expression in *EpCAM*+ cell clusters of 43 tumor sections from 22 patients with TNBC (33), where 26 sections were from 13 treatment-naïve patients and 17 sections were from nine residual TNBCs. Tissue architecture integrity in these sections was confirmed by hematoxylin and eosin staining (left). The color scale represents log-transformed normalized gene expression (red, highest; blue, lowest). Representative spatial mapping revealed a consistent pattern of *EGFR* expression in chemo-naïve and post-NAC-resistant TNBCs, and its spatial relationships with *CCNE1* and *CDK2* co-expression in tumor sections. Venn diagrams illustrate quantitative analyses for the relationships among *EGFR*, *CCNE1*, and *CDK2* co-expression (numbers in black represent the number of spatial clusters for all patients in the cohort, and % marked in red represent the proportion of these spatial clusters within all *EpCAM*+ clusters).

All analyses of scRNA-seq and spatial transcriptomics data were performed using R Statistical Software (version 4.3.1; R Core Team 2023) in RStudio [version 2023.06.0, RStudio Team (2020); http://www.rstudio.com/; RRID:SCR_000432]. Additional packages used were Seurat (version 4.3.0.1; RRID:SCR_007322), ggplot2 (version 3.4.2; RRID:SCR_014601), dplyr (version 1.1.2; RRID:SCR_016708), pheatmap (version 1.0.12; RRID:SCR_016418), and ggvenn (version 0.1.10; RRID:SCR_025300). Codes for Fig. 2A–D are available at Github: https://github.com/rladdach/anti-EGFR-ADC/tree/main.

### Cell culture conditions

All breast cancer and nontumorigenic epithelial cell models were obtained from the KCL Breast Cancer Now Unit or St. John’s Institute of Dermatology. The human monocytic cell line U937 was sourced from ATCC (RRID:CVCL_U937). Human primary epidermal melanocytes were from ATCC (PCS-2000-012), and the human B lymphocyte cell line RPMI8226 (RRID:CVCL_0014) and RPMI8866 (RRID:CVCL_1668) was from ECACC. The cell lines CAL51 (RRID:CVCL_1110), MCF7 (RRID:CVCL_0031), MDA-MB-231 (RRID:CVCL_0062), MDA-MB-468 (RRID:CVCL_0419), and SKBR3 (RRID:CVCL_0033) were maintained in high-glucose DMEM-GlutaMAX with 10% heat-inactivated FCS. The cell lines HCC1143 (RRID:CVCL_1245), HCC1806 (RRID:CVCL_1258), HCC1937 (RRID:CVCL_0290), RPMI8226, T47D (RRID:CVCL_0553), and U937 were cultured in RPMI1640-GlutaMAX with 10% FCS. SUM149 cells (RRID:CVCL_3422) were cultured in F-12 Hams medium supplemented with 5% FCS, 5 μg/mL insulin, and 1 μg/mL hydrocortisone. All reagents were from Thermo Fisher Scientific. MCF10A cells (RRID:CVCL_0598) were cultured in Mammary Epithelial Cell Growth Basal Medium, supplemented with SingleQuots Supplements (both from Lonza). Human primary epidermal melanocytes were cultured in Melanocyte Growth Medium (Cell Applications Inc.). Cell lines were authenticated by short tandem repeat profiling. Cells were used once tested negative for mycoplasma and used up to 30 passages. All cell lines were maintained in a 5% CO_2_-humidified incubator at 37°C.

### siRNA-mediated gene silencing

EGFR-targeting siRNA sequence (sc-29301) was purchased from Santa Cruz Biotechnology. Transient transfection was performed using siRNA Transfection Reagent (sc-29528, Santa Cruz Biotechnology) according to the manufacturer's protocols. The protocol was repeated after 48 hours for a second round of knockdown. EGFR expression was checked with flow cytometry to measure mean fluorescence intensity (MFI).

### Flow cytometric analysis

To detect EGFR protein expression levels in cell lines, cells were detached with trypsin for 3 minutes and direct immunofluorescence staining was performed for 20 minutes on ice using the anti-EGFR IgG1 antibody cetuximab (Merck Serono; RRID:AB_2459632), followed by a goat anti-human IgG FITC secondary antibody (2BScientific; RRID:AB_218360). Samples were acquired using the FACSCanto II flow cytometer equipped with BD FACSDiva Software (BD Biosciences; RRID:SCR_001456), and data were analyzed with FlowJo_V10 software (RRID:SCR_008520) to measure MFI. The Propidium Iodide Flow Cytometry Kit (Abcam) was used to monitor cell cycle progression via flow cytometry according to the manufacturer’s protocol.

### Whole blood peripheral blood mononuclear cell extraction

Peripheral blood samples were collected from healthy volunteers or from the UK National Health System Blood and Transplant system from anonymous donor leukocyte cones. Peripheral blood mononuclear cells (PBMC) were isolated using Ficoll Paque PLUS (GE Healthcare) density gradient centrifugation. PBMCs were re-suspended in freezing medium [50% FCS, 10% dimethyl sulfoxide (Sigma Aldrich)] and stored at −80°C until required for downstream analyses. To detect EGFR protein expression on immune cells, PBMCs were incubated with Human TruStain FcX Fc Receptor Blocking solution (BioLegend), and fluorescently conjugated antibodies: CD3-FITC (clone: OKT3, BioLegend; RRID:AB_1929898), CD14-PE (clone: HCD14, BioLegend; RRID:AB_830678), CD56-BUV395 (clone: NCAM16.2, BD Biosciences; RRID:AB_2687886), CD19-BV421 (clone: SJ25C1, BioLegend; RRID:AB_10897802) to determine immune cell subtypes of T cells, B cells, monocytes, and NK cells. The cells were further stained with human IgG1 isotype control or cetuximab labeled with Alexa Fluor 647 (Alexa Fluor 647 Antibody Labeling Kit, Invitrogen) and analyzed using a flow cytometer.

### ADC production and characterization

Cetuximab IgG1 was stochastically conjugated to the CDK inhibitor SNS-032 payload (Tocris Bioscience) via an MC-Val–Ala-PAB linker (Cambridge Bioscience) to produce cetuximab–SNS-032 ADC. MC-Val–Ala-PAB is a cleavable ADC linker featuring a maleimide group, a Val–Ala dipeptide, and a para-aminobenzyl (PAB) spacer. Maleimide is a thiol-specific covalent linker that forms disulfide bonds with cysteine residues of proteins. Val–Ala-PAB is a protease-cleavable linker that is designed for efficient payload release upon proteolysis by cathepsin.

Briefly, the interchain disulfides of cetuximab were partially reduced with reducing agent TCEP for 90 to 180 minutes. The reduced antibody was diluted with 2 mmol/L EDTA-PBS to 2 mg/mL. Linker–payload was dissolved in 10 mmol/L DMSO. Conjugation of the antibodies was achieved by the addition of an excess of the linker–payload to a 1:1 mixture of the reduced antibody and propylene glycol, at a final protein concentration of 1 mg/mL.

The antibody and the linker–payload were incubated at room temperature at 20°C for 1 hour to form the ADC. The reaction was quenched with an excess of N-acetylmaleimide. The ADC was further diluted 1:1 with PBS 3% cyclodextrin and then bound to a Protein A resin. The resin-bound ADC was washed with PBS 3% cyclodextrin to remove excess small-molecule impurities and then released from the resin. The ADC was formulated through G25 desalting into PBS 3% cyclodextrin and 0.2 µm filtered prior to aliquoting and −80°C storage.

The ADC was characterized using hydrophobicity interaction chromatography (HIC) and size exclusion chromatography (SEC), which provided information on overall purity, drug antibody ratio (DAR), and the degree of aggregate formation. HIC was performed on a TOSOH Butyl-NPR 4.6 mm × 3.5 cm, 2.5-µm column (Tosoh Corp., Japan) at 0.8 mL/minute with a 12-minute linear gradient between mobile phase A [1.5 mol/L (NH_4_)_2_SO_4_, 25 mmol/L NaPi, pH = 6.95 ± 0.05] and mobile phase B [75% 25 mmol/L NaPi, pH 6.95 ± 0.05, 25% isopropyl alcohol]. The sample was loaded up to a maximum loading of 10 µL, and data were collected at 280 and 214 nm; all reported data are 214 nm. The aggregate content of each conjugate preparation was assessed by SEC on a TOSOH TSKgel G3000SWXL 7.8 mm × 30 cm, 5-µm column at 0.5 mL/minute in 10% isopropyl alcohol, 0.2 mol/L potassium phosphate, 0.25 mol/L potassium chloride, pH 6.95. Samples were loaded, and data were collected at 214, 252, and 280 nm. All reported data are at 214 nm. For the determination of protein concentration, the samples were injected at various volumes (0.5, 1, 2, 3, 4, and 5 µL), and the area under the curve at 214 nm was recorded. A linear regression model (area = concentration × slope + intercept) was fitted to the data and used to calculate the concentration of the ADC.

### Surface plasmon resonance analysis of antibody and ADC binding affinity

Surface plasmon resonance binding experiments were performed using a Biacore T200 instrument (GE Healthcare; RRID:SCR_019718). Anti-His tag antibodies were immobilized onto a CM5 sensor chip using an amine-coupling protocol according to the manufacturer’s instructions. His-tagged recombinant EGFR protein was injected at a flow rate of 10 µL/minute for 300 seconds. For binding studies, antibodies in a two-fold dilution series (3–100 nmol/L) were injected at a flow rate of 20 µL/minute for 240 seconds, followed by a dissociation time of 900 seconds. All binding experiments were performed at 25°C in 20 mmol/L 4-(2-hydroxyethyl)-1-piperazineethanesulfonic acid (HEPES) pH 7.4, 150 mmol/L NaCl, 0.005% (v/v) surfactant P20. BIAevaluation (GE Healthcare; RRID:SCR_015936) and Origin 8 (OriginLab) were used to analyze the data.

### *In vitro* cell viability assay

To measure cell viability, 5,000 cells per well were plated in 96-well plates and incubated with cetuximab, isotype ADC, ADC, or free inhibitor for 96 hours at 37°C. Cell viabilities were detected by CellTiter 96 AQueous One Solution Cell Proliferation Assay (Promega) according to the manufacturer’s instructions. Optical absorbance was read on a FLUOstar Omega spectrophotometer (BMG Labtech; RRID:SCR_025024).

### Incucyte live-cell imaging analysis

Antibody and ADC internalization assays were carried out using an Incucyte S3 Zoom Live-Imaging system (Essen Bioscience; RRID:SCR_023147). Images and data were obtained and analyzed using Incucyte S3 software (version 2019A). Cells were plated in colorless FluoroBrite DMEM (ThermoFisher Scientific) with 10% FBS. Incucyte Fabfluor-pH Red Antibody Labeling Dye (Essen Bioscience) was pre-mixed with 10-nmol/L cetuximab, ADC, isotype IgG1, or isotype-ADC controls for 30 minutes before adding to the cells. Phase and red fluorescence images were captured every hour for 24 hours to monitor internalization, whereas phase images were captured every 24 hours for 5 days (or 7 days for 3D spheroid models where cells were embedded in 20 μL of solidified Matrigel) to determine the effects of treatments on cell growth. MDA-MB-468 cells were transduced with a lentiviral expression vector encoding an mCherry fluorescent protein tag (mChery-MDA-MB-468). For co-culture experiments using high and low EGFR-expressing cell lines mixed at a one-to-one ratio, the red fluorescence signal (mCherry-MDA-MB-468) and nonfluorescence phase-contrast images (MCF7 or CAL51) were monitored after 5 days of ADC treatment to determine the bystander killing effects of the ADC on low EGFR-expressing cells.

### Live-cell imaging using confocal microscopy

Cancer cells were treated with 10 nmol/L of cetuximab or ADC pre-labeled with Alexa-Fluor-647 Antibody Labeling Kit (Thermo Fisher Scientific) for 3 or 24 hours. After three washes with PBS, cells were then incubated with BioTracker 560 Orange Lysosome Dye (Sigma-Aldrich) for 30 minutes, followed by 5 minutes of Hoechst 3,342 nucleus dye (ThermoFisher Scientific). The endoplasmic reticulum (ER) was stained using ER-Tracker Green (ThermoFisher Scientific) for 30 minutes. All incubations were conducted at 37°C. Live-cell samples were imaged using Spinning Disk Super-Resolution by Optical Pixel Reassignment (SoRa) confocal microscopy (Nikon Centre, KCL), equipped with an Eclipse Ti-2 inverted microscope and Photometrics Prime 95B sCMOS cameras, with a Yokogawa CSU-W1 dual disk scan head for fast confocal and super-resolution. Images were processed in NIS-Elements and colocalization analysis was conducted using Image J software (RRID:SCR_003070).

### Establishment of patient-derived xenograft models of breast cancer

Adult female patients with breast cancer diagnosed and treated at Guy’s and St Thomas’s Hospitals (London, UK) have consented as part of a noninterventional clinical trial (BTBC study REC no.: 13/LO/1248, IRAS ID 131133). Tumor samples were collected from patients via surgery or biopsies. The samples were anonymized and assigned a KCL number and the presence of tumor material was confirmed by a clinician histopathologist or pathology-trained technician. To generate patient-derived xenograft (PDX) models, tumor fragments (∼2 mm) or single-cell digests were orthotopically implanted into the mammary fat pad of host female NSG mice [NOD SCID gamma NSG; (NOD.Cg-Prkdc^SCID^Il2rg^tm1Wjl^/SzJ; RRID:IMSR_JAX:005557)], with either intact or cleared mammary epithelium. All animal experiments were approved by the King’s College London Institutional Committees on Animal Welfare and in compliance with the United Kingdom Home Office Animals Scientific Procedures Act, 1986. Tumor presence was monitored by palpation and caliper measurement. Tumors were passaged to new host animals (either as tumor chunks or single-cell digests), using either fresh or viably frozen material. Other PDX models were obtained through collaborations with Prof. Ellis and Dr. Li, Washington University in St. Louis (WHIM models; ref. 35); Dr. Lewis, Baylor College of Medicine (BCM models; ref. 36); and Dr. Clarke, University of Manchester (MAN models; ref. 37) and orthotopically implanted into host NSG female mice at KCL as described above. For all models, once-grown tumors were harvested, and formalin-fixed paraffin-embedded (FFPE) blocks were prepared.

### PDX tissue microarray preparation

An FFPE tissue microarray (TMA) was constructed, comprising 38 PDX samples established from a total of 35 patients with breast cancer (28 TNBC, five ER+, and two HER2+ tumors). The TMA consisted of 13 PDXs established from 13 different patients consented under KCL ethics, with two further samples with residual diseases from KCL004 and KCL006; nine BCM PDX models, six WHIM PDX models, and eight MAN PDX models (from seven different patients). The disease characteristics of the patients are summarized in Supplementary Table S2.

Representative areas of the donor PDX blocks were firstly marked on H&E stained sections. TMAs were constructed, using the Beecher manual arrayer (Beecher Instruments, Sun Prairie, WI) with 1-mm diameter Ø from FFPE PDX blocks. TMAs were made in triplicate, mainly from the periphery of the carcinoma and other representative areas.

### IHC analyses of surface EGFR expression

EGFR staining was performed on cell line xenograft FFPE blocks or PDX TMA. Normal human breast glandular epithelium tissue and normal tonsil tissue were also used as controls. FFPE blocks were cut at 4-µm slices onto Leica APEX adhesive slides; slices were air-dried overnight at room temperature and then baked at 60°C for 60 minutes to increase adherence. Heat-induced epitope retrieval was performed for 20 minutes at 97°C in the Agilent Dako PT-module using Target Retrieval solution pH 9 (K800421-2) according to the manufacturer’s instructions. Staining was performed on the Agilent Dako Link48 Autostainer platform; all incubations were carried out at room temperature and all rinses were performed in Dako Wash Buffer solution (Agilent, K800721-2). Endogenous peroxidases were blocked for 5 minutes using Dako REAL peroxidase block (Agilent, S202386-2). A mouse monoclonal anti-EGFR antibody [EGFR.113, NCL-L-EGFR (Leica Biosystems; RRID:AB_563696)] was diluted 1:10 and applied to sections for 60 minutes. Primary antibody was detected with diaminobenzidine chromogen, developed using the Dako FLEX EnVision kit (Agilent, K802301-2) according to the manufacturer’s instructions. Counterstaining was performed onboard for 3 minutes using Dako FLEX Hematoxylin (Agilent, K800821-2). Analyses were performed using conventional microscopy, and digital images were collected by NanoZoomer HT Digital Pathology Scanning System (Hamamatsu; RRID:SCR_021658).

### *In vivo* xenograft treatment studies

All animal work described here was carried out under Home Office PPLs PP9706060 (cell line xenografts) and PF642A32A (PDX). Female NSG mice were obtained commercially from Charles River Laboratories. All animal experiments were approved by the King’s College London Institutional Committee on Animal Welfare, and in compliance with the United Kingdom Home Office Animals Scientific Procedures Act, 1986. For all experiments, 4- to 8-week-old mice were used. Female NSG mice were orthotopically injected into the mammary fat pad with 2 × 10^6^ CAL51 or 1 × 10^6^ MDA-MB-468 cells in 50 μL Matrigel (day 1). Once the tumor was palpable, mice received intravenous injections of vehicle, SNS-032 (5 mg/kg), cetuximab, isotype ADC or ADC (7.5 mg/kg), and weekly injections for 4 weeks. For PDX KCL004, mice were implanted with 2-mm pieces of viably frozen tumor tissue (from a single mother tumor) into a mammary fat pad via a trocar. Once tumors were established, mice were given intravenous injections of isotype ADC/ADC (10 mg/kg) twice. Tumors were measured with calipers and volumes were calculated (π × length × width^2^/6). Mice were terminated before tumors reached ≤525 mm^3^.

### Statistical analyses

GraphPad Prism Software (RRID:SCR_002798) and R Statistical Software (RRID:SCR_000432) were used for statistical analyses. Data were presented as mean ± SEM values of three or more independent experiments. *P* values were determined using a Mann–Whitney U test for gene expression analysis. For other experiments, *P* values were determined by a two-tailed unpaired *t* test with significant *P* values indicated with an asterisk, ^∗^, *P* < 0.05; ^∗∗^, *P* < 0.005; ^∗∗∗^, *P* < 0.0005. Nonsignificant *P* values are marked as NS.

### Data availability

scRNA-seq and spatial transcriptomic data generated here are available on GEO (GSE161529 and GSE210616), whereas processed data are provided in their supplementary files. Other data generated in this study are available upon request from the corresponding author.

## Results

### Gene expression patterns of EGFR reveal associations with basal-like/TNBC diseases and G1/S-phase genes

We investigated whether EGFR may be a potential target for ADC therapy by interrogating gene expression in 6,173 primary tumors from five breast cancer datasets. When specimens were stratified by IHC-defined status, in concordance with previous studies (38, 39), EGFR levels were significantly higher in TNBC than non-TNBC and in basal-like PAM50 molecular subtype (Fig. 1A). In the same cohorts, we also investigated the key G1/S-phase cell cycle regulators, namely, CDK4/6/cyclin D (G1-phase), CDK2/cyclin E (G1/S-phase transition), and CDK2/cyclin A (S-phase), which are activated sequentially. Transcriptional regulators CDK7/cyclin H and CDK9/cyclin T control RNA polymerase II activity, whereas CDK7 also actively phosphorylates the CDK2/cyclin E complex for G1/S-phase progression (40). Dysregulation of these axes can lead to cancer (24, 25). We found significant upregulation of cyclin A, cyclin E, and CDK2 in basal-like/TNBCs (Fig. 1A). Although CDK4/6 are upregulated, their binding partner cyclin D is downregulated. CDK7/cyclin H and CDK9/cyclin T are also downregulated or unchanged in basal-like/TNBCs (Supplementary Fig. S1).

Associations between these cell cycle complexes and EGFR in the tumor microenvironment remain undetermined. Thus, we compared the expression of CDK2 and cyclin A/E between high and low EGFR-expressing specimens based on quartile ranges of expression values. In every cohort, we found higher expression of both cyclins in EGFR-high compared with EGFR-low tumors, whereas CDK2 expression was significantly higher in the EGFR-high group in TCGA (Fig. 1B). Given the dysregulation of CDK2/cyclin A/E axis, CDK2 inhibitors may offer a specific approach as ADC warheads. Moreover, compared with matched pre-treatment samples, *EGFR*, *CCNA1*, *CCNE1*, and *CDK2* genes were retained in post-NAC-resistant TNBCs (*n* = 27; Fig. 1C, although *CCNA1* expression was low across datasets), highlighting that these molecules may be relevant targets in primary as well as in residual disease settings.

A number of ADCs cause treatment-related hematotoxicity reactions, with dose-limiting adverse events such as lymphopenia, neutropenia, and thrombocytopenia, caused by apoptosis of megakaryocyte progenitors or disruption of microtubule function during bone marrow mitosis (41). ADC binding to target antigen-expressing immune cells may increase the potential risk for toxicity. We therefore investigated EGFR expression on human PBMCs by flow cytometric analysis and found negligible expression on the main immune cell types tested (Fig. 1D).

These findings confirm dysregulated expression of CDK2, cyclin A, and cyclin E in basal-like/TNBCs, and co-expression with EGFR-high tumors, including post-NAC-resistant TNBCs.

### scRNA-seq and spatial transcriptomic analyses reveal co-expression of EGFR with CDK2/cyclin E in TNBCs

We next explored transcriptional co-expression of these targets within TNBC tumor architecture using published datasets (32, 33). We applied dimensionality reduction (tSNE) to single-cell transcriptomes of 150,290 cells in 27 treatment-naïve tumors (32). Cell clustering identified two malignant tumor cell populations defined by *EpCAM*+ epithelial cells (Fig. 2A). HER2 expression and ER expression were evaluated as internal controls for the TNBC subtype (Supplementary Fig. S2A and S2B). We identified *EGFR*, *CDK2*, and *CCNE1* expression, although overall *CCNA1* expression was too low and therefore excluded from analysis. Distinct cell clusters of *EGFR*-high and *CCNE1*-high cells were found predominantly in the *EpCAM*+ clusters in TNBC (Fig. 2B), where *EGFR *was expressed in 23.1% ± 1.6% of *EpCAM*+ cells, compared with 4.4% ± 0.7% in HER2+ and 3.5% ± 0.5% in ER+ tumor cells. *CCNE1* was measured in 10.3% ± 0.8% of *EpCAM*+ TNBC, compared with 2.0% ± 0.4% in HER2+, and 2.2% ± 0.2% in ER+ cells. When we investigated *CCNE1* and *CDK2* expression in *EpCAM*+ *EGFR*+ cells (Fig. 2C), we found higher level of co-expression in TNBC (cyclin E: 15.6% ± 1.0% vs. 3.2% ± 1.2% in HER2+ and 3.4% ± 0.5% in ER+ cells; CDK2: 22.5% ± 1.7% vs. 4.4% ± 1.1% in HER2+ and 9.3% ± 1.2% in ER+ cells). Additionally, only *CDK4*, but not *CCND1* or *CDK6*, showed a correlation with *EpCAM*+ *EGFR*+ cells (Supplementary Fig. S3A and S3B).

We next interrogated the transcriptional states of spatially resolved intratumoral cellular populations for their expression of *EGFR*, *CCNE1*, and *CDK2* by spatial transcriptomic analyses (Fig. 2D). We analyzed 43 tumor sections from 22 patients with TNBC (33), including treatment-naïve and post-NAC residual tumors. Tissue architecture is maintained in these sections and provides context for high-dimension transcriptional measurements within *EpCAM*+ clusters. Spatial-mapping revealed a consistent pattern of *EGFR* expression in chemotherapy-naïve patients, and its spatial relationships with *CCNE1* and *CDK2* co-expression in the same cell clusters. These spatial associations were retained in post-NAC-resistant TNBC showing similar levels of co-expression of *EGFR* with *CDK2*/*CCNE1*. Moreover, we measured an increase in *EGFR* expression in post-NAC samples (76.7% of clusters) compared with pre-treatment samples (58.2% of clusters).

Advanced scRNA-seq and spatial molecular profiling in tumor specimens supported EGFR expression and higher expression of CDK2/cyclin E in TNBC compared with other breast cancer types across primary and post-NAC-resistant TNBCs, and higher levels of co-expression and spatial colocalization in TNBCs. These data identified EGFR and CDK2 pathways as potential targets of combined therapy for aggressive TNBCs.

### Generation of cetuximab–SNS-032 ADC and internalization studies

**Figure 3.**
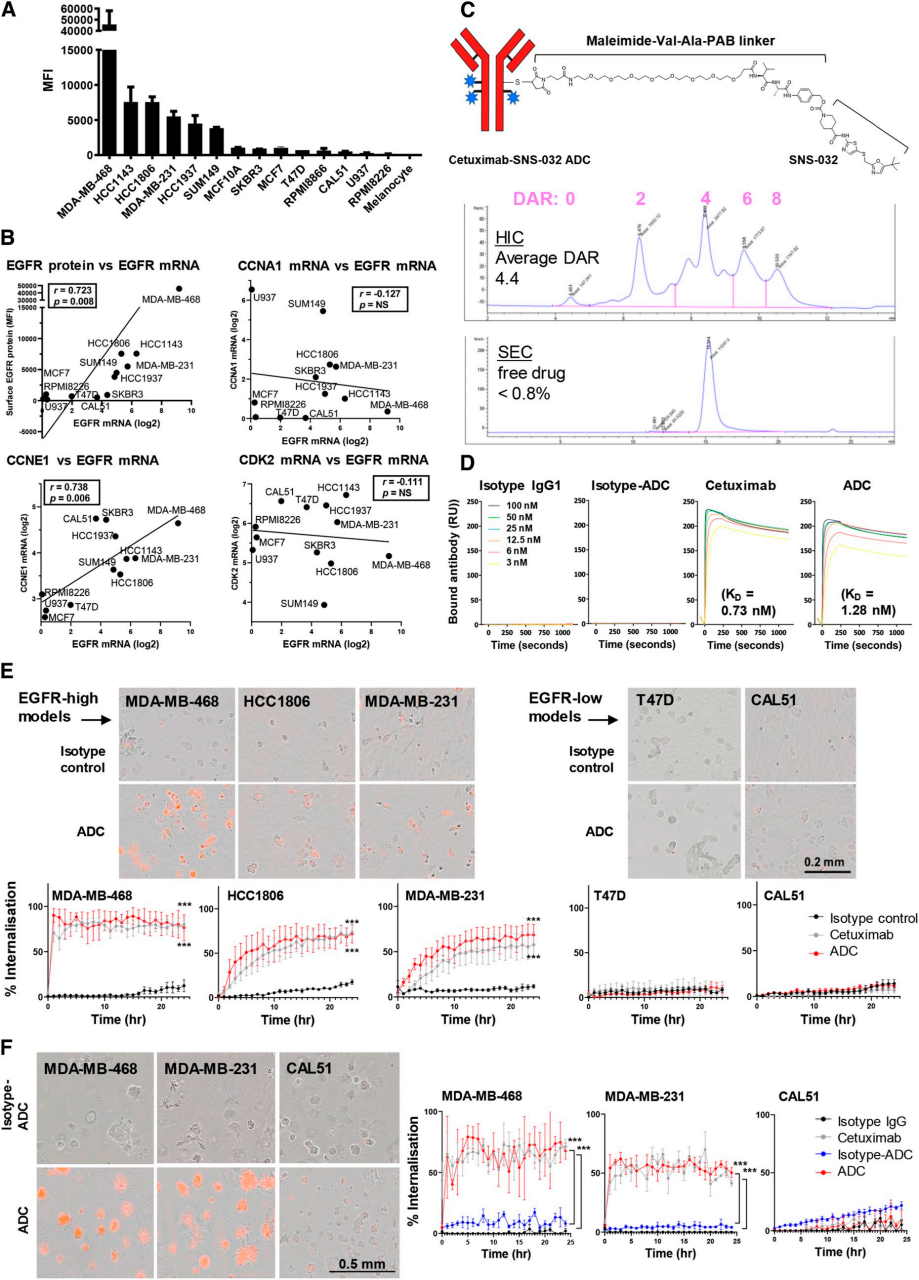
Stochastic conjugation of cetuximab to CDK inhibitor and ADC internalization in live breast cancer cells. **A,** Flow cytometric evaluation of surface EGFR expression (TNBC: MDA-MB-468, HCC1143, HCC1806, MDA-MB-231, HCC1937, SUM149, and CAL51; HER2+: SKBR3; ER+: MCF7, T47D; nontumorigenic epithelial cell model: MCF10A; immune cell model: human B lymphocytes RPMI8866, RPMI8226, and monocytic cell line U937; human primary melanocyte: melanocyte). **B,** EGFR mRNA expression from the Cancer Cell Line Encyclopedia database showed a positive correlation with surface EGFR measured by flow cytometry in **A** (Spearman’s rank coefficient, *r* = 0.723). A high level of correlation was found between EGFR and cyclin E (*r* = 0.738), but not with cyclin A or CDK2. Nonsignificant *P* values are marked as NS. **C,** Top, Schematic diagram of stochastic ADC conjugation by antibody reduction with TCEP and then conjugation to SNS-032 via MC-Val–Ala-PAB. Middle, HIC analysis confirmed an average DAR of 4.4. Bottom, SEC trace indicates negligible ADC aggregation and minimal free linker–payload (less than 0.8%). **D,** Surface plasmon resonance analysis demonstrated similar binding affinity (*K*_*D*_) for cetuximab (0.73 nmol/L) and ADC (1.28 nmol/L). Isotype IgG1 and isotype ADC showed no measurable binding. **E,** Monitoring internalization of Fabfluor-pH-labeled cetuximab, ADC, or isotype control (10 nmol/L) by Incucyte live-cell imaging. Phase and red fluorescence time-course images were captured for 24 hours. Images of internalized antibody display in cytosolic, low pH lysosomal vesicle-associated red fluorescence in cells. Scale bar, 0.2 mm. **F,** Cells were seeded in Matrigel for 5 days, allowing the formation of spheroids. Fabfluor-pH-labeled antibodies or ADC (10 nmol/L) were introduced in the Matrigel and showed rapid internalization in EGFR-high MDA-MB-468 and MDA-MB-231, whereas EGFR-low CAL51 displayed little red fluorescence signals. A low level of internalization was observed for isotype or isotype-ADC controls. Scale bar, 0.5 mm. *P* values determined by two-tailed unpaired *t* test of three independent experiments compared with isotype control.

Despite significant EGFR expression in TNBCs, cetuximab does not engender significant direct cell signaling inhibition against TNBC cells. Based on combined high EGFR and CDK2/cyclin E expression in TNBCs, we aimed to develop an EGFR-targeted approach by developing a cetuximab-based ADC bearing a CDK inhibitor. To identify suitable cellular models, we evaluated EGFR expression in cell lines by flow cytometry using cetuximab (Fig. 3A), and confirmed the correlation of cell surface protein with mRNA expression levels (*r* = 0.723; Fig. 3B). We also found a strong correlation between *EGFR *and *CCNE1* mRNA levels in these cell line models (*r* = 0.738; Fig. 3B) consistent with the correlation data in patient samples (Fig. 1B), but no correlation with *CCNA1* or *CDK2 *expression.

We then generated an ADC by conjugating SNS-032, known to have selective inhibition of CDK2/7/9 over CDK4/6 (25), to cetuximab, through a maleimide-based linker, using the antibody as a vehicle to specifically deliver the inhibitor to cancer cells. HIC analysis confirmed average DAR of 4.4, and SEC analysis indicated negligible ADC aggregation and 0.8% free drug (Fig. 3C). Furthermore, surface plasmon resonance studies showed comparable affinities (*K*_*D*_) of the ADC (1.28 nmol/L) and cetuximab (0.78 nmol/L) to EGFR, consistent with published literature (42), whereas isotype IgG1 and isotype ADC showed no measurable binding (Fig. 3D).

Cetuximab has been reported to internalize into EGFR-expressing cells (43). We aimed to test if the ADC maintained a similar internalization rate after conjugation with the hydrophobic inhibitors. We detected internalization using a pH-sensitive Fabfluor-pH red fluorophore, which displays fluorescence only when sequestered in acidic environments. Incucyte microscopy was employed to enable real-time, kinetic evaluation of internalization. Cetuximab and ADC each displayed comparable and time-dependent increases in cytoplasmic fluorescence, demonstrating EGFR-dependent internalization, with the highest EGFR-expressing model MDA-MB-468 showing rapid internalization rate within the first hour (Fig. 3E). Low levels of uptake were also seen in EGFR-low T47D and CAL51, whereas low levels of target-independent, nonspecific endocytosis of the isotype control antibody were also observed in all cell lines in later time points. In concordance with data from the corresponding monolayer cultures, ADC internalization rates in MDA-MB-468 and MDA-MB-231 spheroids were comparable to cetuximab and higher than isotype controls (Fig. 3F).

Our data show that cetuximab and the corresponding cetuximab-ADC demonstrated similar cell binding properties and EGFR-dependent internalization into acidic compartments of tumor cells.

### Spatiotemporal analysis of ADC internalization in lysosomal clusters and inhibition of cell cycle

The primary purpose of the ADC is to increase the tumor selectivity and thus relative efficacy versus toxicity of cytotoxic treatments by releasing the payload within the acidic and protease-rich late lysosomal compartment inside tumor cells. Having shown cytoplasmic ADC uptake with low-resolution live-cell imaging, we next monitored the intracellular uptake and fate of the ADC using super-resolution confocal microscopy. Alexa-Fluor-647-labeled ADC (magenta signal) was shown to associate with MDA-MB-468 cell membranes within 15 minutes of time-lapse imaging, then observed to bud inward into endosomes and transported into the cytoplasm. By 45 minutes, the fluorescence signal accumulated intracellularly, although the signal on the surface membrane weakened, suggesting ADC endocytosis (Supplementary Fig. S4A).

**Figure 4.**
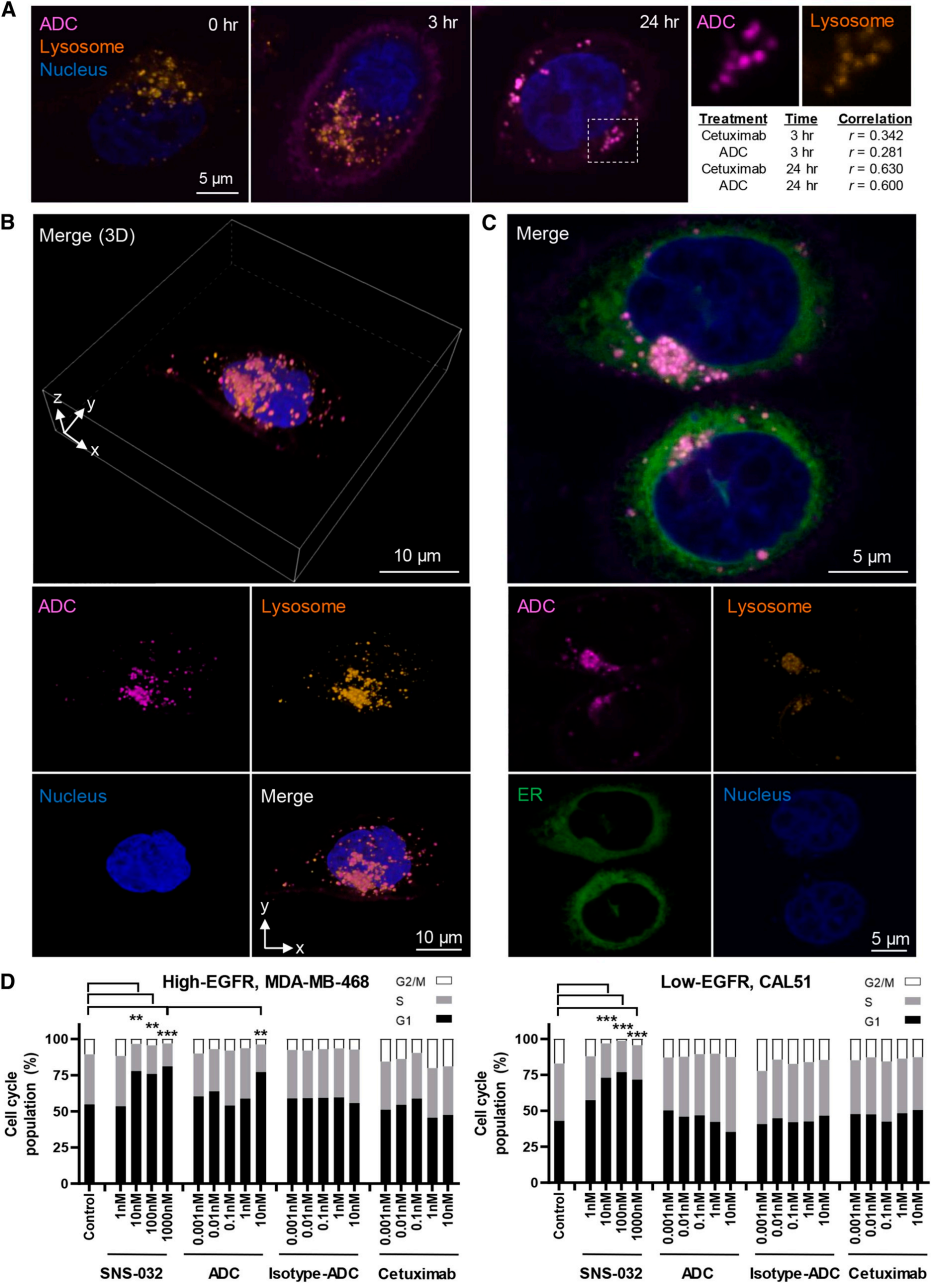
Spatiotemporal analysis of ADC internalization in lysosomal clusters and inhibition of cell cycle progression. **A,** Images represent monitoring treatment of MDA-MB-468 with 10 nmol/L Alexa-Fluor-647-labeled ADC (magenta) for 0, 3, and 24 hours and the colocalization with lysosomes. Following incubation, live cells were stained with low pH lysosome dye (orange) followed by Hoechst 3,342 (blue). Scale bar, 5 μm. Very little cell surface binding and uptake of ADC were shown by EGFR-low CAL51 cells at any time point (Supplementary Fig. S4B). Image J colocalization analysis demonstrated moderate colocalization with low pH lysosomes after 3 hours in MDA-MB-468 (cetuximab vs. lysosome, Pearson’s correlation *r* = 0.342, *n* = 45 cells; ADC vs. lysosome, *r* = 0.281, *n* = 34 cells), whereas correlation was strong at 24 hours (cetuximab vs. lysosome, *r* = 0.630, *n* = 64 cells; ADC vs. lysosome, *r* = 0.600, *n* = 28 cells). Right, white box: zoom-in images showing individual channels of a representative area of colocalization between ADC and lysosomal staining. **B,** 3D reconstruction image of a live MDA-MB-468 cell. The Z-stack images showed spatial information of ADC colocalization within lysosome clusters at 24 hours. Top, merged Z-stack 3D image; bottom, 2D images of individual channels. Scale bar, 10 μm. 3D reconstruction of the Z-stack images demonstrating internalized ADC-lysosome clusters can be found in Supplementary Video S1. **C,** Confocal images showing a high level of colocalization of internalized ADC in lysosome clusters within the ER in close proximity to the nucleus. Scale bar, 5 μm. **D,** Quantitative analyses on the distribution of cell cycle phases by flow cytometry after 72 hours of cetuximab-ADC treatment, compared with SNS-032, isotype-ADC, and unconjugated-cetuximab controls. Significant cell cycle inhibition (G1 arrest) was observed with SNS-032 (at 10, 100, 1,000 nmol/L) in both TNBC models, whereas only high EGFR-expressing MDA-MB-468 demonstrated significant inhibition by the ADC (10 nmol/L), but not in EGFR-low CAL51. *P* values determined via the *χ*^2^ test against untreated control in three independent experiments.

The efficacy of this ADC relies on the internalization and release of payloads within acidic intracellular compartments via linker cleaved by proteases in late lysosomes. After demonstrating internalization within an hour in live MDA-MB-468 cells, we investigated if this ADC colocalizes with lysosomes at later stages. Cells were treated with Alexa-Fluor-647-labeled cetuximab/ADC for 3 and 24 hours. BioTracker Lysosome Dye (orange) was used to visualize lysosomes (pH 5.0). High-resolution imaging allowed visualization of individual organelles that contained the ADC (Fig. 4A). Very low surface binding and ADC uptake were shown by EGFR-low CAL51 (Supplementary Fig. S4B). In MDA-MB-468, correlation analysis showed weak colocalization of cetuximab with lysosomes at 3 hours (*r* = 0.342) or ADC (*r* = 0.281), indicating that the internalized antibodies were within early endosomes but not yet located in acidic compartments for payload release. After 24 hours, we found a strong correlation of lysosome markers with both cetuximab (*r* = 0.630) and ADC (*r* = 0.600). Late lysosomes contain mature cysteine proteases; hence, conjugated cytotoxic payloads are expected to be released at this final stage of endocytosis. 3D reconstruction of Z-stack images confirmed spatial colocalization of ADC in individual vesicles within lysosome clusters (Fig. 4B; Supplementary Video S1). We further demonstrated that the ADC predominantly accumulated in late lysosomes located in the ER adjacent to the nucleus (Fig. 4C).

These data demonstrate ADC internalization and colocalization within the endosomal and later within the low pH and protease-rich late lysosomal compartment, a critical attribute required for the effective release of the payload. Our findings suggest similar kinetic patterns for ADC and cetuximab and indicate that conjugation with CDK inhibitors did not affect the antibody’s capacity for endocytosis and late lysosomal localization for payload release.

The ADC payload SNS-032 can be a potent selective inhibitor targeting the CDK2/cyclin A/cyclin E complexes and inhibit G1/S-phase progression (25). Treatment with SNS-032 (10, 100, and 1,000 nmol/L) significantly induced G1-phase arrest in MDA-MB-468 and CAL51 (Fig. 4D) and proportionally decreased S-phase and G2/M-phase compared with untreated controls. The ADC induced G1-phase arrest in EGFR-high MDA-MB-468 at 10 nmol/L but not in EGFR-low CAL51, indicating specific effects of the ADC. Consistent with the reported resistance of these TNBC cells to anti-EGFR inhibition (17, 21), unconjugated cetuximab (and isotype ADC) showed no significant effects on cell cycle distribution at the dosages tested. These findings confirm that the ADC can inhibit cell cycle progression.

### ADC restricts cell viability and growth and exhibits bystander killing effects

**Figure 5.**
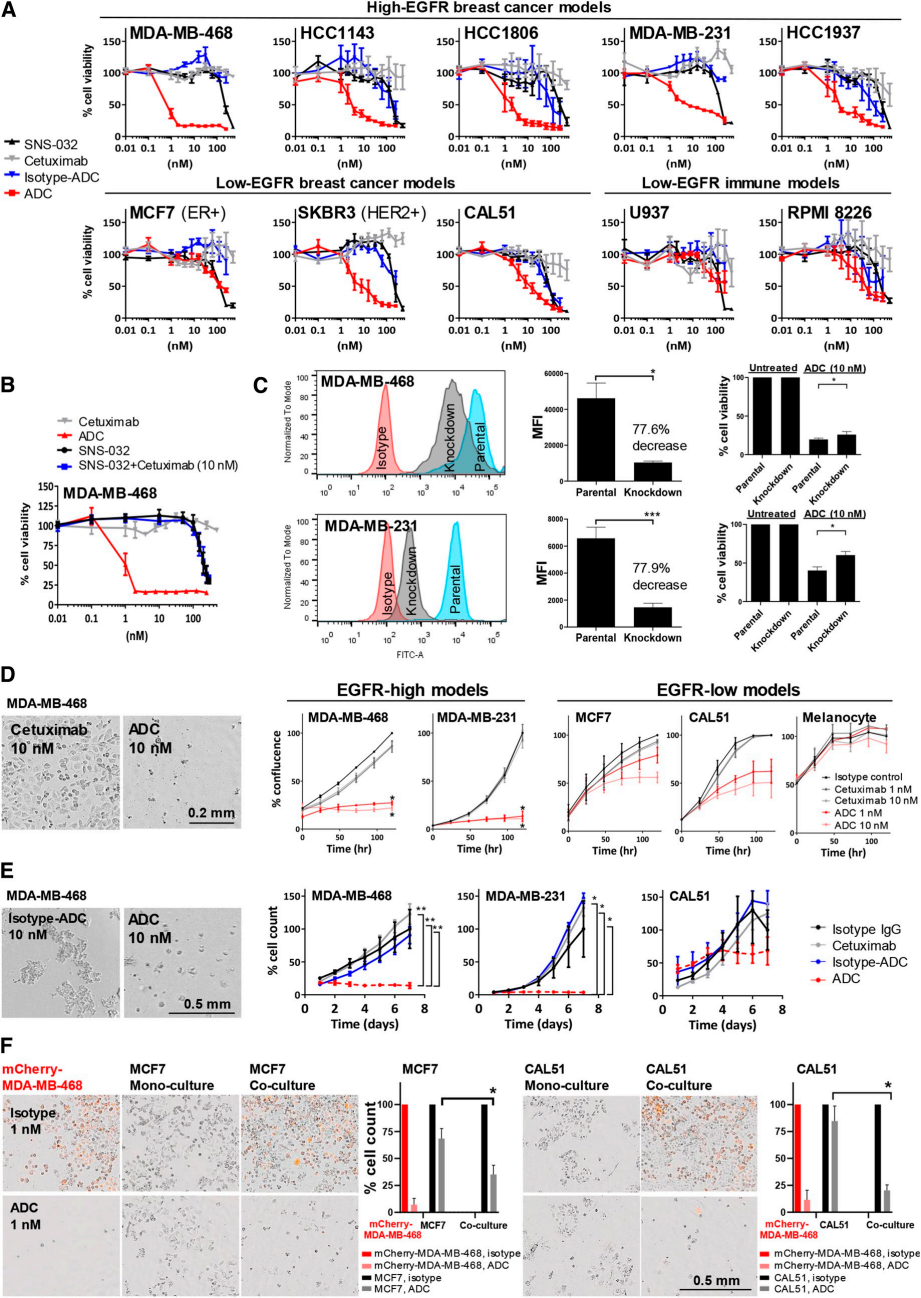
Anti-EGFR ADC reduces breast cancer cell activities and demonstrated bystander killing effects. **A,** MTT viability assay following 96-hour treatment with SNS-032, cetuximab, isotype ADC or ADC. Top, EGFR-high breast cancer models. Bottom, EGFR-low breast cancer and immune cell models. **B,** Cell viability assessment of MDA-MB-468 showed that the addition of SNS-032 did not re-sensitize cells to EGFR inhibition by cetuximab alone, whereas reduced cancer cell viability was detected only when SNS-032 was conjugated to cetuximab as an ADC, suggesting that inhibition of cancer cell viability was induced by ADC internalization and subsequent drug release within cancer cells. SNS-032 efficacy improved by conjugating in the ADC instead of treating it as a free drug. **C,** Reduction of surface EGFR expression using siRNA, and cell viability comparisons between parental and knockdown cells after 96 hours of ADC treatment (10 nmol/L). **D,** Time-lapse measurement of cell confluency for cells treated with isotype control (10 nmol/L), cetuximab, and ADC (1 or 10 nmol/L) using Incucyte live-cell microscopy (representative phase images of EGFR-high MDA-MB-468). Scale bar, 0.2 mm. **E,** TNBC spheroids in Matrigel were treated with 10 nmol/L cetuximab, ADC, or isotype controls, and confluence measured for 7 days using Incucyte. ADC-treated MDA-MB-231 and MDA-MB-468 showed reduced spheroid growth, whereas the ADC showed less potent effects on CAL51. Scale bar, 0.5 mm. **F,** MDA-MB-468 were transduced with a lentiviral expression vector encoding a mCherry fluorescent protein tag (mChery-MDA-MB-468). Bystander killing effects of the ADC were accessed in co-cultures of high and low EGFR-expressing cells in a one-to-one ratio. High EGFR-expressing mCherry-MDA-MB-468 and either low EGFR-expressing MCF7 or CAL51 cells were plated alone as mono-culture or co-culture, and treated with 1 nmol/L ADC or unconjugated-isotype control antibody. Cell count was measured by Incucyte after washing at 120 hours. Scale bar, 0.5 mm. *P* values determined by two-tailed unpaired *t* test of three independent experiments.

We next evaluated whether the ADC could selectively kill EGFR-expressing cells. Despite comparable binding properties between cetuximab and ADC, all 10 cell lines tested in viability assays were largely resistant to cetuximab (gray line, up to 500 nmol/L), regardless of EGFR expression (Fig. 5A). Isotype ADC (blue line) exerted cytotoxicity after 96 hours, only with IC50 much higher than cetuximab-ADC (red line; MDA-MB-468 IC50 of ADC = 0.79 nmol/L, isotype ADC or cetuximab ≥10,000 nmol/L; MDA-MB-231 IC50 of ADC = 6.72 nmol/L, isotype ADC = 3,805 nmol/L; cetuximab was unmeasurable). The IC50 of SNS-032 (black line) ranged from 94.6 to 370.8 nmol/L, consistent with a previous report (25). We furthermore observed modest on-target ADC activity in some low EGFR-expressing (CAL51 and SKBR3) cells. Furthermore, treatment with cetuximab plus free SNS-032 at similar molar concentrations to those in the ADC did not potentiate any anticancer effects above those of free inhibitor alone, and further viability reduction was only detected when SNS-032 was conjugated in an ADC format (Fig. 5B). The efficacy of SNS-032 was thus improved by conjugating the inhibitor to cetuximab as an ADC, rather than by additional treatment with free drug (IC50 in MDA-MB-468: ADC = 0.79 nmol/L, SNS-032 = 309 nmol/L, SNS-032 + cetuximab = 299 nmol/L). These data suggest that inhibition was most likely induced by ADC internalization and subsequent drug release, but not through any inhibitor-potentiated direct Fab-mediated cell signaling effect by cetuximab.

We generated EGFR-knockdown cells showing a >77% decrease in EGFR expression (MFI). Compared with parental cells, EGFR-knockdown cells showed reduced sensitivity to ADC (Fig. 5C), confirming that the ADC was performed in an antigen-specific manner.

We next measured cell growth by Incucyte time-lapse imaging for 120 hours (Fig. 5D). The ADC impacted the growth of MDA-MB-468 (% confluency: 1 nmol/L, 27% ± 2%; 10 nmol/L, 22% ± 3%) and MDA-MB-231 (1 nmol/L, 13% ± 5%; 10 nmol/L, 11% ± 3%) compared with isotype control–treated cells. In contrast, at the same dose range unconjugated–cetuximab showed little effect (minimum 86% confluency). The ADC showed partial growth inhibition in EGFR-low models MCF7 (1 nmol/L, 79% ± 8%; 10 nmol/L, 56% ± 6%) and CAL51 (1 nmol/L, 62% ± 13%; 10 nmol/L, 51% ± 16%). Inhibition effects on nonmalignant primary human melanocytes that do not express EGFR were minimal (1 nmol/L, 108% ± 1%; 10 nmol/L, 92% ± 9%; Fig. 3A, flow cytometry). Monitoring spheroid growth in Matrigel for 7 days showed significant inhibition of MDA-MB-468 and MDA-MB-231 growth by the ADC but not by isotype ADC (Fig. 5E). There was some on-target ADC uptake in EGFR-low CAL51, but growth inhibition effects of the ADC were not significantly higher than controls.

In addition to EGFR-expressing cancer cells being targeted, bystander killing effects may contribute to ADC efficacy through the release of the membrane-permeable payloads from the internalizing cell following linker cleavage or by payload release following cancer cell death. The payloads could then be taken up by neighboring cells that do not express sufficient EGFR levels, as a potential mechanism based on our observations of EGFR and CDK2/cyclin E co-expression and spatial colocalization in TNBC. To interrogate the potential of bystander killing effects, we developed co-cultures of mCherry-transfected MDA-MB-468 (red) with EGFR-low MCF7/CAL51 (colorless) cells. In mono-cultures, ADC treatment (1 nmol/L) reduced mCherry-MDA-MB-468 cell count to <25% compared with control-treated wells after 120 hours, whereas inhibition effects were small in MCF7 and CAL51 (Fig. 5F). However, the ADC exhibited significant bystander killing on MCF7 and CAL51 when each was co-cultured with mCherry-MDA-MB-468: MCF7 cell count reduced to 35.1% ± 13.3% (68.5% ± 14.3% in mono-culture); CAL51 reduced to 20.2% ± 8.1% (84.6% ± 21.7% in mono-culture).

These data suggest that cetuximab–SNS-032 ADC can impair EGFR-expressing cell and spheroid growth and exert bystander cytotoxicity of neighboring EGFR-low cancer cells.

### ADC restricts orthotopic xenograft growth

**Figure 6.**
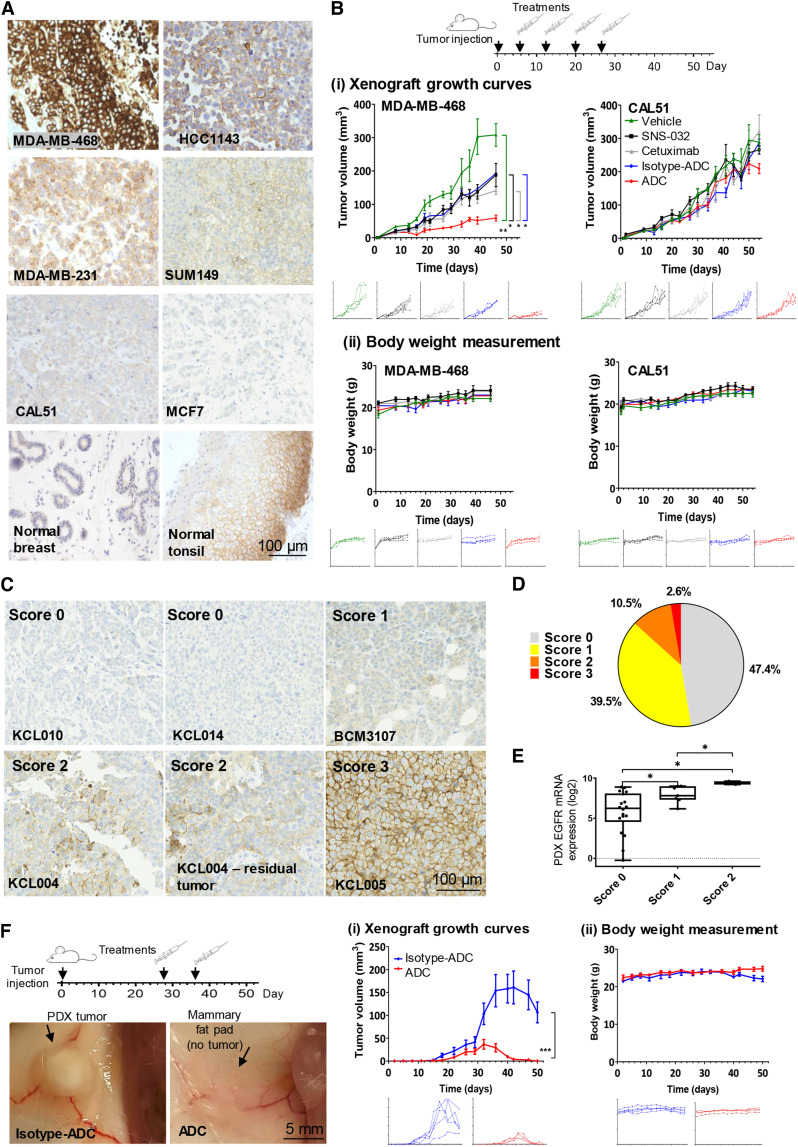
ADC growth inhibition of orthotopic TNBC xenografts *in vivo.***A,** Staining for EGFR was confirmed using FFPE blocks of cell line xenografts of known EGFR expression pattern (see Fig. 3A; EGFR-high/positive: MDA-MB-468, HCC1143, MDA-MB-231, SUM149; EGFR-low: CAL51, MCF7), compared with human normal breast glandular epithelium and normal tonsil. Scale bar, 100 μm. **B,** Effects of ADC treatment on tumor growth *in vivo*. (i) Orthotopic tumor growth of MDA-MB-468 and CAL51 xenografts (*n* = 4) treated with vehicle, SNS-032 (5 mg/kg), cetuximab, isotype ADC, or ADC (7.5 mg/kg, weekly injection for 4 weeks). *P* values determined by one-way ANOVA. (ii) Body weight measurement. **C,** IHC evaluation of EGFR in a TMA of 38 PDX samples established from 35 patients (28 TNBC, five ER+, and two HER2+; disease characteristics: Supplementary Table S2). Representative images showing various staining intensities across samples (scale bar, 100 μm). Pathologists scored the TMA spots for EGFR positivity: score 0, no membrane staining; score 1, weak incomplete membrane staining in >10% cells; score 2, moderate complete membrane staining in >10% cells or strong complete membrane staining in ≤10% cells; score 3, strong (intense and uniform) complete membrane staining in >10% cells. **D,** Pie chart showing out of 38 PDX samples: 47.4% (*n* = 18, gray) were negative for membrane EGFR expression; 39.5% (*n* = 15, yellow) were score 1; 10.5% (*n* = 4, orange) were score 2; 2.6% (*n* = 1, red) is highly positive (score 3). **E,** Microarray-based EGFR mRNA expression was compared with membrane EGFR staining positivity tested by IHC. *P* value determined by two-tailed unpaired *t* test. **F,** Effects of ADC treatment on KCL004 PDX tumor growth *in vivo*. Left, representative tumor images after two doses of isotype ADC or ADC, where no visible residual tumor was found in any mice treated with ADC during the experimental timeframe. (i) KCL004 PDX orthotopic tumor growth (*n* = 5) with isotype-ADC or ADC treatment (two doses, 10 mg/kg). (ii) Body weight measurements. *P* values determined by one-way ANOVA.

We next evaluated ADC effects in orthotopic xenografts grown in the mouse mammary fat pad to partly recapitulate the complexity of human disease. EGFR expression is a prerequisite for EGFR-specific ADC therapy. We confirmed EGFR expression in paraffin-embedded xenografts (Fig. 6A), in line with FACS evaluations (Fig. 3A). In concordance with the Human Protein Atlas dataset (proteinatlas.org, accessed on February 2024; ref. 44), we found restricted EGFR expression in normal human breast glandular epithelium and specific staining in the stratified squamous compartment of human tonsil. We measured significant tumor growth delay in ADC-treated mice (day 46: 58.8 ± 8.7 mm^3^) compared with vehicle (308.1 ± 34.12 mm^3^), SNS-032 (187.9 ± 34.7 mm^3^), cetuximab (141.1 ± 11.2 mm^3^), or isotype ADC (193.0 ± 3.4 mm^3^) for the cetuximab-resistant MDA-MB-468 xenografts (Fig. 6Bi). The ADC effect was notable considering that the conjugated SNS-032 dose measured only a molar fraction (1.65%) of that of the free uncoupled inhibitor treatment. None of the treatments induced weight loss (Fig. 6Bii) or any signs of overt toxicity. Meanwhile, the ADC did not exert any significant tumor restriction on EGFR-low CAL51 xenografts compared with the same controls.

To select a patient-derived model for efficacy studies, EGFR expression was examined by IHC on a TMA consisting of 38 PDX samples established from 35 patients (28 TNBC, five ER+, and two HER2+ tumors). Surface EGFR staining was detected in 52.6% of the PDX [score 1: 39.5% (*n* = 15); score 2: 10.5% (*n* = 4); score 3: 2.6% (*n* = 1; Fig. 6C and D)]. PDX surface EGFR scores correlated with corresponding mRNA expression in the same samples (Fig. 6E). KCL004 has a score of 2 EGFR expression when grown as a PDX. Moreover, when previously treated *in vivo* with olaparib, this xenograft subsequently showed resistance, giving rise to residual disease which retained EGFR expression levels (Fig. 6C). Two ADC doses of 10 mg/kg given to animals with established parental KCL004 tumors greatly inhibited xenograft growth (Fig. 6Fi). Xenografts in all five mice reduced in size after ADC treatment, and no palpable residual tumor was re-formed in the timeframe of the experiment (day 50: isotype ADC: 106.4 ± 22.7 mm^3^; ADC: 0 ± 0 mm^3^). ADC treatment did not induce weight loss (Fig. 6Fii).

Our findings demonstrate the therapeutic potential of the ADC in xenograft models. Despite bearing only a fraction of the payloads compared with the free drug administered, ADC treatment was significantly more potent than inhibitor alone. These studies suggest that the ADC restricts TNBC tumor growth, and may benefit patients with treatment-resistant disease.

## Discussion

By selecting a cell cycle–targeted inhibitor, and cetuximab to direct the payload to EGFR-expressing cancer cells, we generated cetuximab–SNS-032 ADC, bearing a small fraction of the drug alone dose required to engender antitumor effects, and we evaluated this as a treatment against TNBC. This ADC restricted EGFR-expressing cancer cell growth *in vitro* and in xenografts while showing safe *in vivo* administration and low effects against EGFR-low breast cancer and immune cell models.

There are 12 ADCs approved for solid tumors and hematological malignancies, with over 100 ADCs at various stages of clinical testing, reflecting a fast-rising interest. However, approved ADCs are broadly based on two classes of payload, tubulin inhibitors (DM1, DM4, MMAE, and MMAF) that disrupt microtubule formation in the cytosol, and DNA-interactive damaging agents (calicheamicin, DXd, SN38, and PBD; ref. 20). Here, we test proof of concept for an ADC strategy, using a cell cycle inhibitor for targets identified based on the frequency of combination of a target surface antigen expression and druggable oncogenic pathway in selected patient cohorts.

Cetuximab binds to the extracellular domain of EGFR with higher affinity than its ligands (EGF, TGFα, amphiregulin, and epiregulin) and is designed to block the autophosphorylation of its tyrosine kinase–dependent signaling pathway (12). Despite evidence of therapeutic efficacy in other cancers, anti-EGFR antibodies have shown limited activity in TNBCs that are not exclusively dependent on EGFR signaling for survival (17, 21). An alternative ADC approach that does not depend on antibody-mediated inhibition of EGFR signaling could help re-define EGFR as a therapeutic target for patient populations who do not respond to cetuximab.

EGFR levels alongside those of CDK2 and its cyclin partners were upregulated in primary basal-like/TNBCs. Furthermore, both CDK2 cyclin partners were expressed at higher levels in EGFR-high tumors. Additionally, we demonstrated co-expression and colocalization of EGFR and the cell cycle genes in the tumor microenvironment by scRNA-seq and spatial transcriptomic analyses. These prompted the study of EGFR as a potential target using an ADC coupled with CDK inhibitors. Cyclin E and EGFR have previously been reported to be actionable targets in neoadjuvant chemotherapy–resistant TNBCs (45). This is consistent with our findings in matched baseline and post-NAC-resistant tumors, and in spatial transcriptomic analyses, in both of which we demonstrated that residual TNBCs retained EGFR and cell cycle gene expression. Patients with residual disease have a high risk of developing metastatic disease and limited treatment options other than palliative chemotherapy. Therefore, further investigation of EGFR status could point to patient subgroups who could potentially receive anti-EGFR ADC linked to a CDK inhibitor. However, structural homology between CDK proteins renders the identification of CDK2 inhibitors that do not possess some affinity for other kinases very challenging. In the absence of a highly specific CDK2 inhibitor, SNS-032 is reported to be potent against CDK2 (46) but also to inhibit RNA polymerase II activity by targeting CDK7/9. However, alongside their binding partners, CDK7/9 is often downregulated in basal-like/TNBCs; hence, TNBCs may not rely on CDK7/9 activity. Moreover, the molecular structure of SNS-032 contains a piperidine group, where its amine bridge (–NH) can react with the dipeptide linker for linker–payload synthesis, rendering this compound a suitable candidate for conjugation.

We generated the ADC by conjugating cetuximab with SNS-032 via thiol–maleimide reaction, using the antibody as a vehicle to specifically deliver the payload to cancer cells. The Val–Ala dipeptide has been employed in the approved loncastuximab–tesirine ADC against B-cell lymphoma (47). This linker is readily cleavable by cysteine cathepsins in lysosomes while remaining reasonably stable in plasma, of interest for the design of this ADC for which localization in lysosomal compartments was an important attribute. Moreover, overexpression of lysosomal proteases is frequently found in breast cancers (48), and this offers another advantage for therapies using cathepsin-sensitive ADCs. Using super-resolution confocal microscopy, it was possible to visualize individual intracellular vesicles and to obtain spatiotemporal information on ADC-lysosome colocalization. Lysosomes are dynamic organelles with high mobility driven by motor proteins (49) that travel throughout the cell in response to nutrient levels and lipid distribution in membranes, whereas a relatively immobile perinuclear lysosome pool forms near the ER, where it controls the directional transportation of lipid cholesterol, protein, and in this case, cleaved payloads from the ADCs. In our study it was necessary to stain only the acidic lysosomes, as any other organelles (early/late endosomes) often contain pre-mature environments that are less acidic, with pre-mature cathepsins, and are distant from ER/nucleus, and thus likely insufficient for payload release. Our live-cell spatiotemporal data showed a high level of colocalization of the internalized ADC in lysosome clusters within ER and in close proximity to the nucleus, an attribute crucial for drug release with the cleavable linkers.

The ADC, but not cetuximab alone, could induce cell cycle arrest and engender cytotoxic functions specifically against EGFR-high tumor cells, with subsequent release of free payloads to trigger bystander cytotoxicity against neighboring EGFR-low cells in the heterogenic tumor microenvironments.

Despite the *in vivo* administered ADC carrying only a small molar fraction (1.65%) of the free SNS-032, the tumor-restricting effects of the ADC were superior to the much higher SNS-032 doses required to exert tumor growth restriction. Anti-EGFR ADCs carrying conventional payloads have thus far shown manageable safety profiles in clinical trials (22, 23). Consistent with these, we observed no weight loss or signs of overt toxicity with ADC administration *in vivo*. Furthermore, *in vitro* assays showed no/low cytotoxic effects of the ADC on human cutaneous melanocytes or immune cells which, if targeted, could result in myelosuppression. However, cutaneous toxicity is possible through the ADC targeting low/medium EGFR-expressing nonmalignant skin tissues (50), and this should be monitored and managed in the clinic.

In conclusion, our study introduces an ADC strategy based on analyses of the frequency of the combination of a target surface antigen expression and druggable oncogenic signaling activity in selected patient cohorts. Cetuximab–SNS-032 ADC inhibited the cell cycle, restricted cellular growth and viability, exerted bystander killing effects on local EGFR-low cancer cells, and despite carrying a small fraction of the inhibitor dose needed to exert antitumor effects, the ADC inhibited orthotopic TNBC xenograft growth *in vivo*. This ADC strategy could potentially be considered for further research to treat EGFR-expressing patients with limited treatment options and unfavorable prognoses.

## Supplementary Material

Supplementary Table 1 Supplementary Table 1. Summary of transcriptomic datasets from different patient cohorts.

Supplementary Table 2 Supplementary Table 2. Disease characteristics of breast cancer patients for PDX TMA samples (please see Figure 6C–6F).

Supplementary Figure 1 Supplementary Figure 1. Expression of G1/S-phase cell cycle genes and transcriptional regulators with basal-like/TNBCs.

Supplementary Figure 2 Supplementary Figure 2. Single-cell RNA sequencing analyses of HER2, ER and EGFR in breast cancer.

Supplementary Figure 3 Supplementary Figure 3. Single-cell RNA sequencing analysis of G1/S-phase cell cycle genes in breast cancer.

Supplementary Figure 4 Supplementary Figure 4. Live-cell confocal microscopy of ADC internalization.

Supplementary Video 1 Supplementary Video 1. Confocal Z-stack 3D video of ADC colocalization within lysosome clusters. Confocal microscopy video of a 3D reconstruction image of a live MDA-MB-468 cell showing spatial information of ADC colocalization within lysosome clusters after 24 hr of treatment (please also see Figure 4B). Cell represents treatment of 10 nM Alexa-Fluor-647 labelled ADC (magenta) for 24 hr, and stained with lysosome dye (orange) followed by Hoechst 3342 nucleus dye (blue).
