# Supplementary material for: Anti-EGFR Antibody–Drug Conjugate Carrying an Inhibitor Targeting CDK Restricts Triple-Negative Breast Cancer Growth
Source: Clin Cancer Res. 2024 May 21;30(15):3298–315. doi: 10.1158/1078-0432.CCR-23-3110 (PMC11292198; doi:10.1158/1078-0432.CCR-23-3110)
Supplement: Supplementary Table 1 — Summary of transcriptomic datasets from different patient cohorts. [file ccr-23-3110_supplementary_table_1_suppst1.pdf]

Cohorts for Figure 1

**Transcriptomic data – Primary breast cancers**<br>*Guy's*<br>*SCAN-B*<br>*METABRIC*<br>*TCGA breast cancer*<br>*ICGC*<br>References [26-30]	**Samples**:<br>Total primary breast cancer samples, *n* = 6173:<br>Guy's: primary TNBC, *n* = 131; HER2+, *n* = 32; ER+, *n* = 14;<br>SCAN-B: primary TNBC, *n* = 165; HER2+, *n* = 420; ER+, *n* = 2425;<br>METABRIC: primary TNBC, *n* = 101; HER2+, *n* = 117; ER+, *n* = 347;<br>TCGA: primary TNBC, *n* = 112; HER2+, *n* = 158; ER+, *n* = 426;<br>ICGC: primary TNBC, *n* = 73; HER2+, *n* = 4; ER+, *n* = 182<br>**(Figure 1A, 1B; Supplementary Figure 1)**
**Transcriptomic data – Post-neoadjuvant chemotherapy–resistant residual TNBCs**<br>*Matched baseline-residual TNBC cohort*<br>References [31]	**Samples:**<br>Total matched primary and residual TNBC samples, *n* = 27 of which:<br>King's College London: (*n* = 8);<br>Royal Marsden Hospital (*n* = 9);<br>The Netherlands Cancer Institute (*n* = 10).<br>**(Figure 1C)**

Cohorts for Figure 2

**Single-cell RNA sequencing data:**<br>*scRNA-seq cohort*<br>References [32]	**Samples:**<br>Total samples, *n* = 27 primary samples, 150290 cells, of which:<br>TNBC (*n* = 8 samples, 54819 cells);<br>HER2+ (*n* = 6 samples, 31917 cells);<br>ER+ (*n* = 13 samples, 63554 cells).<br>**(Figure 2A, 2B, 2C; Supplementary Figure 2A, 2B, 3A, 3B)**
**Spatial transcriptomic data:**<br>*Spatial transcriptomic cohort*<br>References [33]	**Samples:**<br>TNBC samples, *n* = 22 patients, with 43 tumor sections of which:<br>Primary (pre-treatment), *n* = 13 patients with 26 tumor sections;<br>Residual (post-NAC), *n* = 9 patients with 17 tumor sections.<br>**(Figure 2D)**

Cohort for Figure 3

**Transcriptomic data - cell line:**<br>*Cancer Cell Line Encyclopedia (CCLE)*<br>(https://portals.broadinstitute.org/ccle)	**Samples:**<br>CCLE database was used to characterize mRNA expression of EGFR, cyclin A, cyclin E and CDK2 in human cell lines.<br>**(Figure 3B)**

Supplementary Table 1. Summary of transcriptomic datasets from different patient cohorts.
