# Supplementary material for: Anti-EGFR Antibody–Drug Conjugate Carrying an Inhibitor Targeting CDK Restricts Triple-Negative Breast Cancer Growth
Source: Clin Cancer Res. 2024 May 21;30(15):3298–315. doi: 10.1158/1078-0432.CCR-23-3110 (PMC11292198; doi:10.1158/1078-0432.CCR-23-3110)
Supplement: Supplementary Table 2 — Disease characteristics of breast cancer patients for PDX TMA samples (please see Figure 6C–6F). [file ccr-23-3110_supplementary_table_2_suppst2.pdf]

**Gender (*n* = 35)**	
Female<br>Male	35 (100.0%)<br>0 (0.0%)
**Cancer type (*n* = 35)**	
TNBC	28 (80.0%)
ER+	5 (14.3%)
HER2+	2 (5.7%)
**Treatment naïve (Yes/No) (*n* = 35)**	
Yes	14 (40.0%)
No	21 (60.0%)
**Tumor site (*n* = 35)**	
Primary	26 (74.3%)
Local recurrence	1 (2.9%)
Lymph node	4 (11.4%)
Pleural effusion	1 (2.9%)
Ascites	1 (2.9%)
Metastatic breast cancer	2 (5.7%)
**TMA PDX sample (*n* = 38)**	
Directly from patient	36 (95.0%) (two samples taken from MAN10 patient)
PDX residual disease after olaparib treatment	2 (5.0%)

Supplementary Table 2. Disease characteristics of breast cancer patients for PDX TMA samples (please see Figure 6C-6F).
