# Supplementary material for: Anti-EGFR Antibody–Drug Conjugate Carrying an Inhibitor Targeting CDK Restricts Triple-Negative Breast Cancer Growth
Source: Clin Cancer Res. 2024 May 21;30(15):3298–315. doi: 10.1158/1078-0432.CCR-23-3110 (PMC11292198; doi:10.1158/1078-0432.CCR-23-3110)
Supplement: Supplementary Figure 1 — Expression of G1/S-phase cell cycle genes and transcriptional regulators with basal-like/TNBCs. [file ccr-23-3110_supplementary_figure_1_suppsf1.pdf]

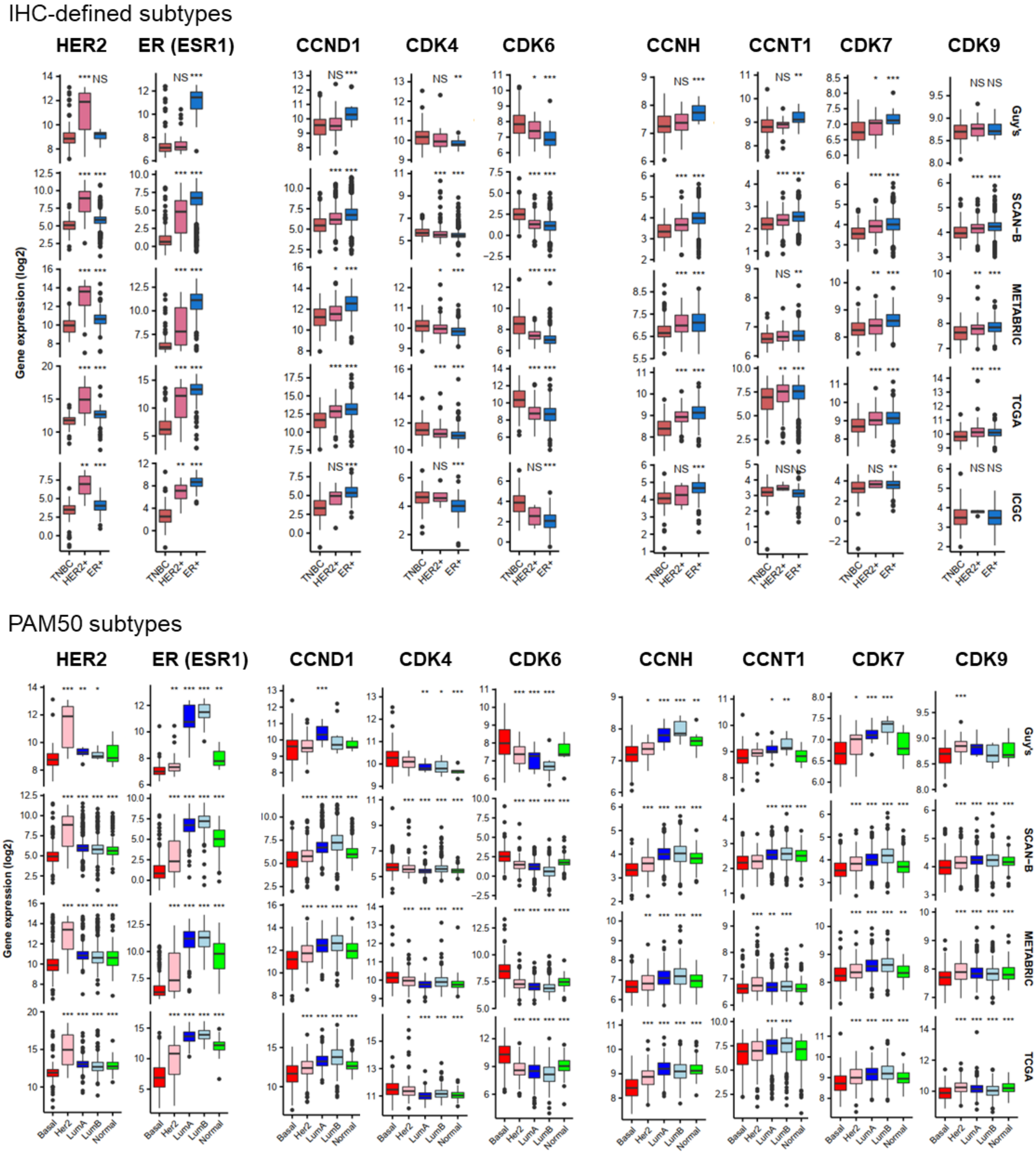

Supplementary Figure 1. Expression of G1/S-phase cell cycle genes and transcriptional regulators with basal-like/TNBCs.

The expression of HER2 and ER (ESR1) were evaluated as internal controls for the TNBC subtype. Gene expression analysis of G1/S-phase cell cycle genes cyclin D (CCND1), CDK4, CDK6 and transcriptional regulators CDK7/cyclin H (CCNH) and CDK9/cyclin T (CCNT1) were stratified according to their IHC-defined (top panels) and PAM50 (bottom panels) subtypes (see Figure 1). All *P*-values are compared against the TNBC or Basal-like subtype.
