# Supplementary material for: Anti-EGFR Antibody–Drug Conjugate Carrying an Inhibitor Targeting CDK Restricts Triple-Negative Breast Cancer Growth
Source: Clin Cancer Res. 2024 May 21;30(15):3298–315. doi: 10.1158/1078-0432.CCR-23-3110 (PMC11292198; doi:10.1158/1078-0432.CCR-23-3110)
Supplement: Supplementary Figure 2 — Single-cell RNA sequencing analyses of HER2, ER and EGFR in breast cancer. [file ccr-23-3110_supplementary_figure_2_suppsf2.pdf]

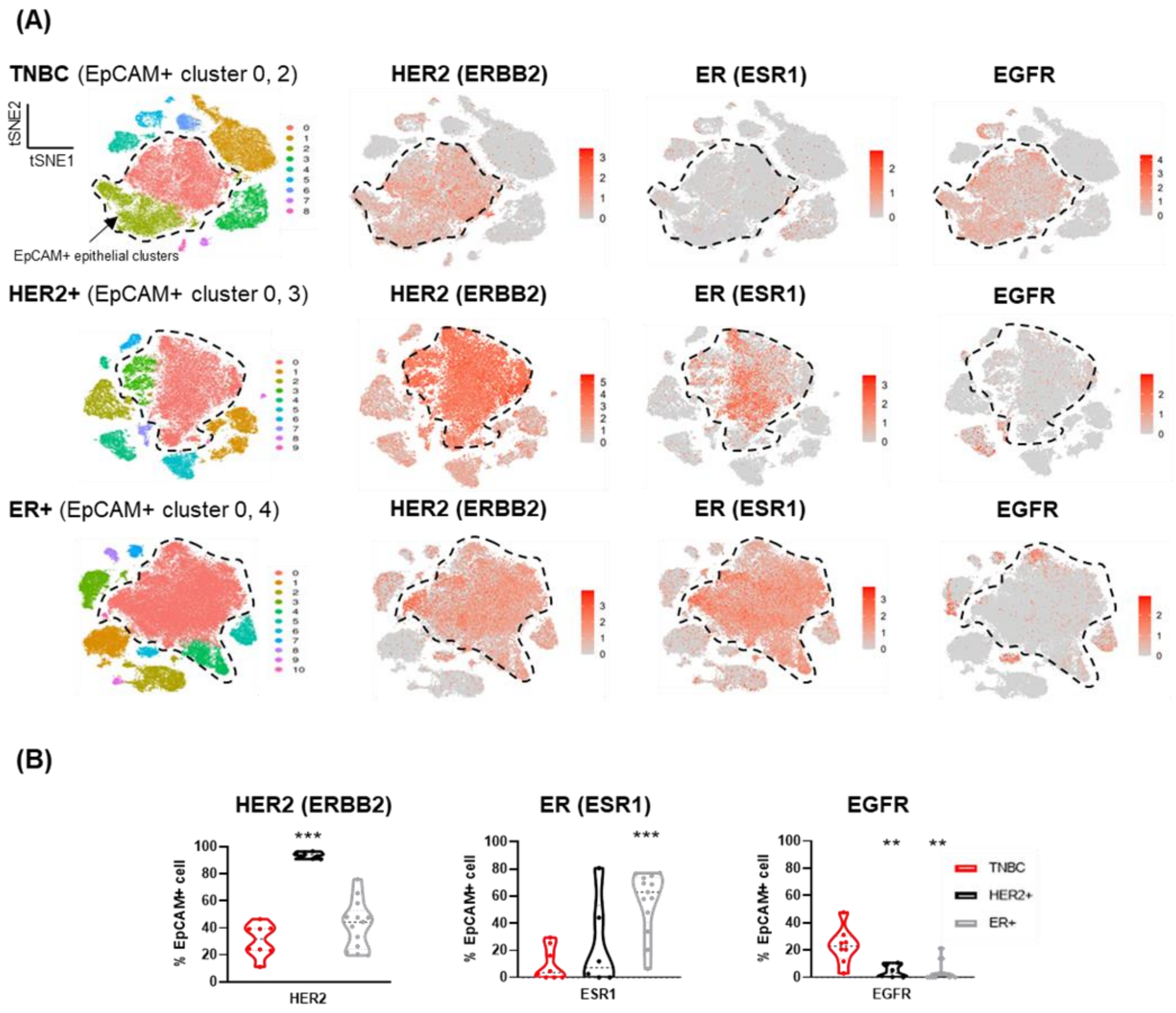

Supplementary Figure 2. Single-cell RNA sequencing analyses of HER2, ER and EGFR in breast cancer.

(A) Dimensionality reduction t-SNE map of combined scRNA-seq transcriptomes of total 150290 cells from 27 untreated primary tumors (TNBC, *n*=8 samples, 54819 cells; HER2+, *n*=6 samples, 31917 cells; ER+, *n*=13 samples, 63554 cells) colored by cell cluster (see Figure 2A and 2B). **(B)** In the EpCAM+ cells**,** the expression of HER2 (ERBB2) and ER (ESR1) were evaluated as internal controls for the TNBC subtype. EGFR is only upregulated in EpCAM+ TNBCs, but not in the HER2+ or ER+ samples. *P*-values determined by two-tailed unpaired *t* test against TNBC subtype.
