# Supplementary material for: Anti-EGFR Antibody–Drug Conjugate Carrying an Inhibitor Targeting CDK Restricts Triple-Negative Breast Cancer Growth
Source: Clin Cancer Res. 2024 May 21;30(15):3298–315. doi: 10.1158/1078-0432.CCR-23-3110 (PMC11292198; doi:10.1158/1078-0432.CCR-23-3110)
Supplement: Supplementary Figure 3 — Single-cell RNA sequencing analysis of G1/S-phase cell cycle genes in breast cancer. [file ccr-23-3110_supplementary_figure_3_suppsf3.pdf]

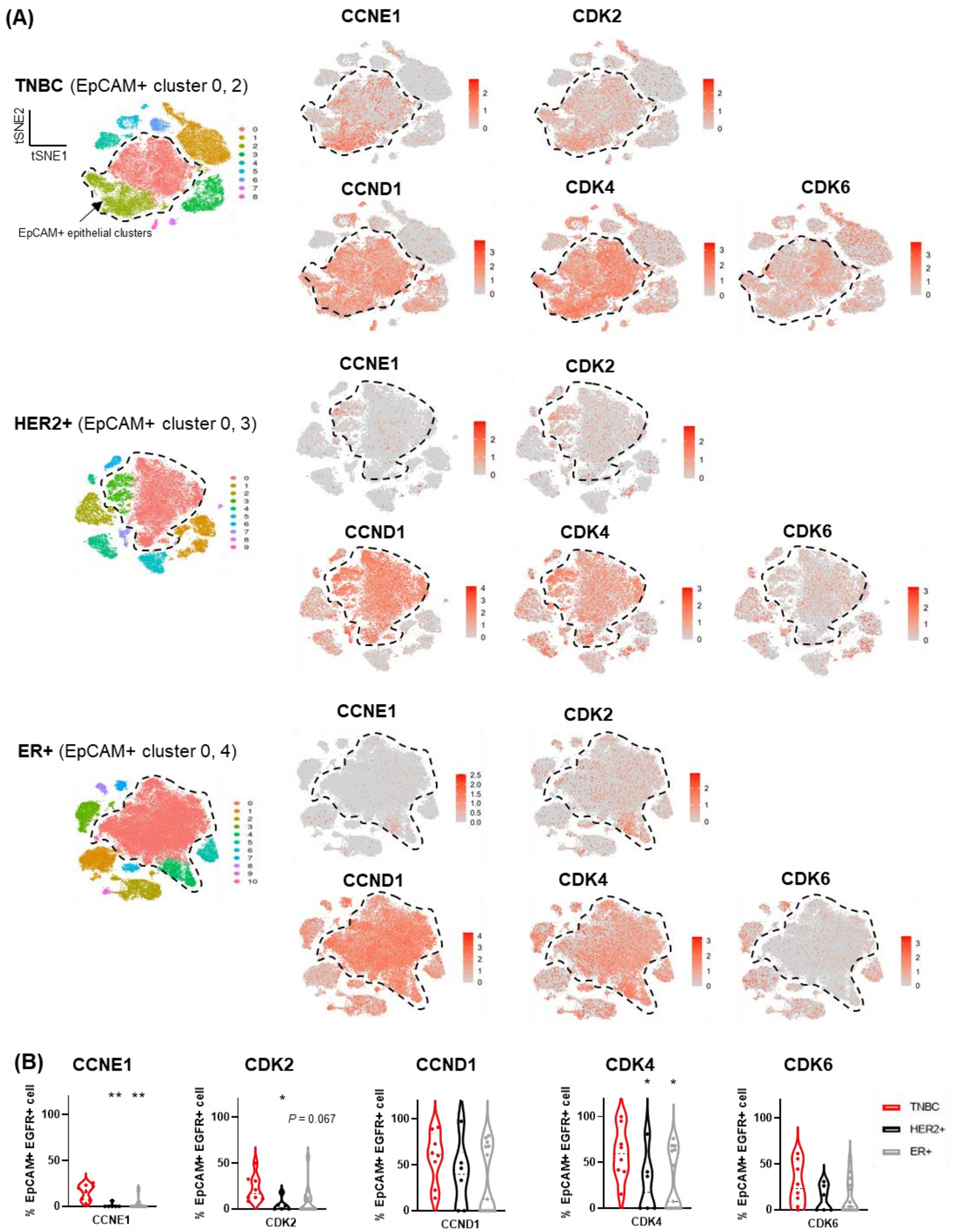

Supplementary Figure 3. Single-cell RNA sequencing analysis of G1/S-phase cell cycle genes in breast cancer.

(A) Dimensionality reduction t-SNE maps of G1/S phase CDKs and cyclins expression in EpCAM+EGFR+ cells, and **(B)** analyses demonstrated CDK2 and cyclin E are upregulated in TNBCs (see Figure 2A and 2B). Although CDK4 is upregulated, its binding partners (CDK6 and cyclin D) are not in upregulated in TNBCs. *P*-values determined by two-tailed unpaired *t* test against TNBC subtype.
