# Supplementary material for: Anti-EGFR Antibody–Drug Conjugate Carrying an Inhibitor Targeting CDK Restricts Triple-Negative Breast Cancer Growth
Source: Clin Cancer Res. 2024 May 21;30(15):3298–315. doi: 10.1158/1078-0432.CCR-23-3110 (PMC11292198; doi:10.1158/1078-0432.CCR-23-3110)
Supplement: Supplementary Figure 4 — Live-cell confocal microscopy of ADC internalization. [file ccr-23-3110_supplementary_figure_4_suppsf4.pdf]

(A)

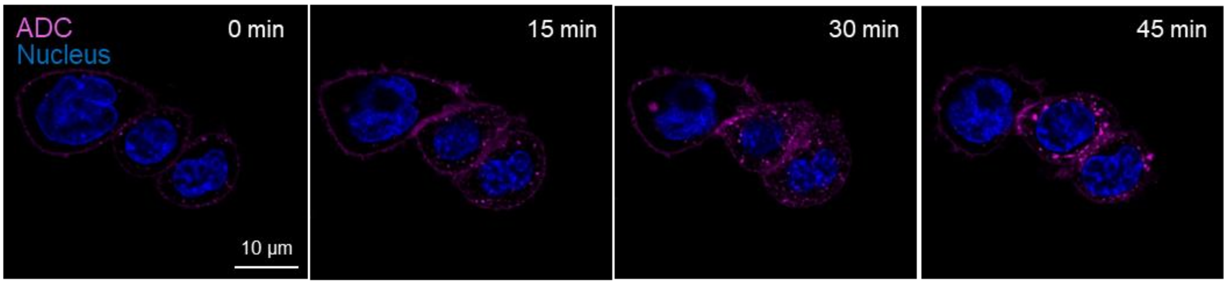

(B)

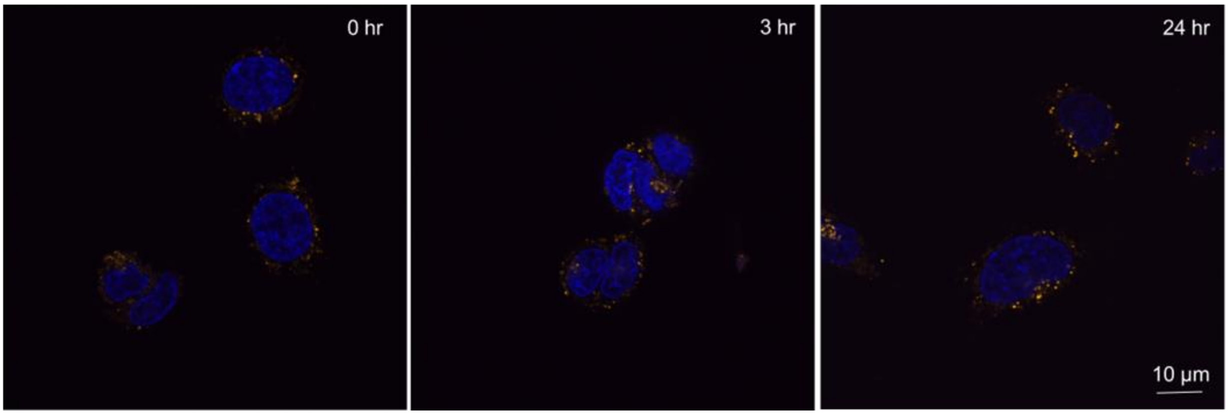

Supplementary Figure 4. Live-cell confocal microscopy of ADC internalization.

(A) Live MDA-MB-468 cells were incubated with Hoechst 3342 nuclear dye (blue) and treated with 10 nM Alexa-Fluor-647-labeled ADC (magenta). Time-lapse fluorescence microscope image sequences were tracked using super-resolution confocal microscopy for the times indicated and showed surface binding and endocytosis within 45 mins. Scale bar, 10 µm. **(B)** Cell images represent treatment of 10 nM Alexa-Fluor-647 labelled ADC (magenta) for 0, 3 and 24 hr. Live CAL51 cells were then stained with low pH lysosome dye (orange) followed by Hoechst 3342 nucleus dye (blue). Scale bar 10 µm. Very low cell surface binding and uptake of ADC was shown by the low EGFR-expressing CAL51 cells at any time-point, compared to high ADC uptake shown in MDA-MB-468 (see Figure 4A).
